# Arginine methylation of HSPA8 by PRMT9 inhibits ferroptosis to accelerate hepatitis B virus-associated hepatocellular carcinoma progression

**DOI:** 10.1186/s12967-023-04408-9

**Published:** 2023-09-15

**Authors:** Wensheng Deng, Jiaoyu Ai, Wanlin Zhang, Zhenyu Zhou, Muqi Li, Likun Yan, Lidong Zhang, Zongjing Huang, Ziyi Wu, Junhua Ai, Hai Jiang

**Affiliations:** 1 Department of General Surgery, The First Affiliated Hospital of Nanchang University, No. 17 Yongwaizheng Street, Donghu District, Nanchang City, 330000 Jiangxi China; 2 Department of Gastroenterology, The First Affiliated Hospital of Nanchang University, Nanchang, 330000 Jiangxi China; 3 Department of Clinical Laboratory, Ningbo Yinzhou No. 2 Hospital Ningbo Urology and Nephtology Hospital, Ningbo, 315100 Zhejiang China; 4 Department of Hepatobiliary Surgery, Sun Yat-Sen Memorial Hospital, Sun Yat-Sen University, Guangzhou, 510120 China

**Keywords:** Hepatitis B virus X, Hepatocellular carcinoma, Ferroptosis, Arginine methylation, PRMT9, HSPA8, CD44

## Abstract

**Background:**

The hepatitis B virus X (HBx) protein is an established cause of hepatitis B virus (HBV)-induced hepatocellular carcinoma (HCC). Whether arginine methylation regulates ferroptosis involved in HBx-induced HCC progression has not been reported. This study aimed to explore whether HBx-regulated protein arginine methyltransferase 9 (PRMT9) mediates the involvement of ferroptosis in the development of HCC.

**Methods and results:**

HBx inhibited ferroptosis through promoting PRMT9 expression in HCC cells. PRMT9 suppressed ferroptosis to accelerate HCC progression in vivo. PRMT9 targeted HSPA8 and enhanced arginine methylation of HSPA8 at R76 and R100 to regulate ferroptosis in HCC. HSPA8 overexpression altered the transcriptome profile of HepG2 cells, in particular, ferroptosis and immune-related pathways were significantly enriched by differentially expressed genes, including CD44. HSPA8 overexpression up-regulated CD44 expression and knockdown of CD44 significantly reversed the inhibition of ferroptosis caused by PRMT9 overexpression.

**Conclusions:**

In conclusion, HBx/PRMT9/HSPA8/CD44 axis is a vital signal pathway regulating ferroptosis in HCC cells. This study provides new opportunities and targets for the treatment of HBV-induced HCC.

**Graphical Abstract:**

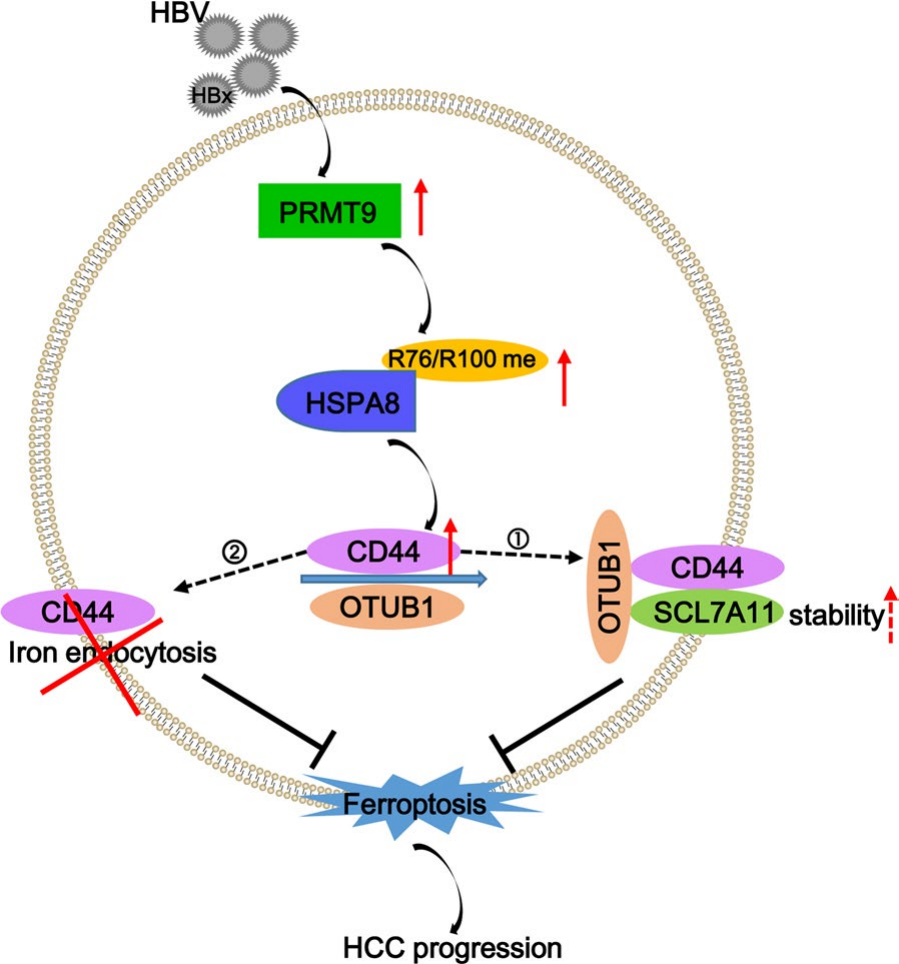

**Supplementary Information:**

The online version contains supplementary material available at 10.1186/s12967-023-04408-9.

## Introduction

Liver cancer is the second most common cause of cancer-related deaths in humans; the main type of primary liver cancer is hepatocellular carcinoma (HCC), which accounts for 85 to 90 percent of all liver cancer cases [1]. Epidemiological studies have shown that chronic hepatitis B virus (HBV) infection is a major risk factor for liver cancer, with HBV infection accounting for more than 50% of all HCC cases worldwide [2]. The hepatitis B virus X (HBx) protein is encoded by the X open reading frame of HBV. HBx plays a critical role in liver cancer and has been reported to regulate the expression of many genes and epigenetic modifications that lead to dysregulation of various pathways and processes in HCC, or to act as a transcriptional activator interacting with nuclear transcription factors and regulating cytoplasmic signaling pathways, such as the Wnt signaling pathway [3]. Therefore, exploring the molecular mechanism of HBx protein regulation is clinically important for the understanding and treatment of HCC caused by HBV infection.

Ferroptosis is a novel form of cell death caused by an imbalance in intracellular lipid peroxidation, which is reflected in biochemical indicators such as accumulation of lipid peroxidation, reactive oxygen species (ROS), Fe^2+^, and malondialdehyde (MDA) [4]. Studies have shown that targeting ferroptosis is an effective way to treat HCC [5]. Currently, there are only two studies related to HBx and ferroptosis. Liu et al. showed that HBx promotes D-GalN-induced ferroptosis in primary mouse hepatocytes through inhibition of SLC7A11 expression by EZH2 [6]. Interestingly, in hepatic stellate cells, HBx exerts an inhibitory effect on ferroptosis [7]. However, the specific role of HBx in the regulation of ferroptosis in HCC remains large unclear and still needs to be further explored.

Protein arginine methylation is a widespread post-translational modification that affects many cellular processes, including transcription, RNA splicing, DNA repair and cell signaling [8]. Protein arginine methylation, a post-translational modification, is catalyzed by nine protein arginine methyltransferases (PRMTs) to participate cancer development. For example, arginine methylation of MDH1 by PRMT4 inhibits glutamine metabolism resulting in suppression of pancreatic cancer [9]. Arginine and lysine methylation of MRPS23 by PRMT7 controls mitochondrial oxidative phosphorylation leading to accelerating breast cancer metastasis [10]. Moreover, PRMT9, also called F-box only protein 11 (FBXO11), plays an important role in HCC [11]. Our previous study showed that PRMT9 is highly expressed in HCC and closely associated with patient survival, and the up-regulation of PRMT9 in HCC is closely related to hepatitis B virus antigen [12], suggesting that high PRMT9 expression in HCC may be associated with HBV infection and that PRMT9 expression may be regulated by the HBx protein. However, whether PRMT9-mediated arginine methylation is involved in HCC via ferroptosis has not been reported.

This study aims to investigate whether HBx-regulated PRMT9 expression mediates the involvement of ferroptosis in the development of HCC through in vivo and in vitro experimental models. The present study will provide new insights into the molecular mechanisms of HCC progression due to HBx from the perspective of PRMT9 arginine methylation.

## Method and materials

### Cell culture and transfection

Human HCC cell line HepG2, MHCC97H, and Huh7 were purchased from Procell and cultured in DMEM (10-013-CV, Corning, China) + 10% FBS (10099-141, GIBCO, China) + 1% P/S at 37 ℃ in an atmosphere containing 5% CO_2_.

In the present study, to overexpress of HBx, PRMT9, and HSPA8 in vitro, the protein coding sequence were cloned into pCDNA3.1 vector, and the blank vector was served as control. Interference of PRMT9 or CD44 expression in vitro was achieved by utilizing siRNA sequences specifically targeting the 3'UTR sequence of PRMT9 or CD44. To knockdown of PRMT9 in vivo, shRNA sequence (as shown in Additional file 5: Table S1) targeted to PRMT9 was cloned into lentiviral vector pSLenti-U6-shRNA-CMV-EGFP-F2A-Puro-WPRE and then packaged into virus to infect MHCC97H cells, and a 2 μg/mL puromycin treatment was performed to harvest a PRMT9 knockdown stable cell line.

For transfection, dilute the plasmid DNA or siRNA (5 μL) in OPTI-MEM (45 μL) and separately dilute the Lipofectamine 2000 reagent in OPTI-MEM (5 μL: 45 μL). Then, gently mixed the diluted Lipofectamine 2000 reagent with the diluted DNA, incubated the mixture at room temperature for 20 min to form the complexes. Cells were seeded in a culture plate the day before the transfection. Transfected the cells when cells at 60–80% confluency. Added the DNA-Lipofectamine 2000 complexes to the cells and incubated at 37 °C in a CO_2_ incubator for 4 h. Finally, after the incubation period, removed the transfection mixture and replace it with fresh culture medium.

### Real-time quantitative PCR (RT-qPCR)

TRIzol reagent (Thermo) was utilized to isolate total RNA from cells. The concentration and purity of total RNA were detected by microspectrophotometer, and the integrity of RNA was detected by agarose gel electrophoresis. Next, total RNA was reverse transcribed to cDNA by RevertAid First Strand cDNA Synthesis Kit (#K1622, Thermo, USA). The cDNA was subjected to qPCR using SYBR Premix Ex Taq™ II Kit (Takara, Dalian, China) on the BIO-RAD CFX96 (Bio-Rad Laboratories, Hercules, CA, USA). GAPDH was served as housekeeping gene to normalized gene expression using 2^−ΔΔCT^ method. The primers used in this study were described in Additional file 5: Table S1.

### Western blotting (WB)

Total protein was extracted by RIPA lysis buffer (P0013, Beyotime) from cells. The concentration of the total protein was detected by the BCA kit (Bio-Rad, Hercules, CA, USA). Approximately 20 μg protein was separated by 12% SDS-PAGE and then transferred onto a PVDF membrane followed by blocking with 5% skimmed milk at 25 °C for 2 h. After that, PVDF membrane was incubated with primary antibodies overnight at 4 °C. Primary antibodies: HBx (1:1000, ab2741, Abcam), PRMT9 (1:1000, PA5-48942, Invitrogen), FTH1 (1: 1000, ab65080, Abcam), GPX4 (1:1000, sc-166570, Santa Cruz Biotechnology), 4-HNE (1: 1000, ab46545, Abcam), HSPA8 (1: 4000, 10654-1-AP, Proteintech), MMA (1:1000, 8015S, CST), sDMA (1:1000, 13222S, CST), GAPDH (1:2000, 60004-1-Lg, Proteintech). Next, the PVDF membranes were incubated with secondary antibodies: Goat Anti-Mouse IgG H&L(HRP) (1: 1000, ab205719, Abcam) and Goat Anti-Rabbit IgG H&L(HRP) (1: 20000, ab6721, Abcam) for 2 h at 25 °C. The bands were exposed to ECL reagent and photographed by Chemiluminometer (Clinx Science Instruments, Shanghai, China) and quantified by Image J software.

### CCK8 assay

CCK8 (Beyotime) assay was used to measure viability of cell. HCC cells were seeded in 96-well plates with a density at 1 × 10^4^ cell/well in 100 μL culture medium. At each time point (0 h, 24 h, 48 h, 72 h, and 96 h), 10 μL of the CCK-8 solution was added into each well and incubated for another 1 h at 37 °C. Finally, the absorbance was measured at 450 nm using a microplate reader (Infinite M1000, TECAN).

### ROS detection

The ROS content in HCC cells was determined by DCFH-DA (CA1410, Solarbio). HCC cells were seeded in 12-well plates with a density at 5 × 10^4^ cell/well. Diluted DCFH-DA agents with serum-free culture medium at a ratio of 1:2000 to a final concentration of 5 μmol/L. Added 500 μL of diluted-DCFH-DA per well and incubated for 10 min at 37 °C. The cells were washed three times to fully remove any DCFH-DA that had not entered the cells. Finally, cells were photographed by a fluorescence microscopy (Olympus Corporation, Japan).

### MDA

The MDA content in HCC cells was determined by MDA detection kit (S0131, Beyotime). HCC cells were lysed by RIPA lysis buffer (P0013, Beyotime) and centrifuged at 10,000*g* for 10 min to harvest supernatant. Added 0.2 mL of MDA assay working solution to 0.1 mL of supernatant sample, mixed and heated in a boiling water bath for 15 min. After cooling to room temperature, sample was centrifuged at 1000*g* for 10 min at room temperature. Finally, the absorbance was measured at 532 nm using a microplate reader (Infinite M1000, TECAN).

### Animal experiments

A total of 25 female 6-week-old BALB/c nude mice were purchased from SPF (Beijing) Biotechnology Co., Ltd. All animal experiments were approved by the Medical Research Ethics Committee of the First Affiliated Hospital of Nanchang University [2020 Medical Research Ethics Review No. (1-52)]. All mice were maintained in a constant temperature SPF facility at 23 °C with free access to food and water. To construct mouse xenograft tumor models, 5 × 10^7^ (in 200 μL) HepG2 cells (stably overexpression of PRMT9) or MHCC97H cells (lentiviral stably knockdown of PRMT9) were injected subcutaneously on the axilla of mice under anesthesia using 50 mg/kg pentobarbital sodium. Starting on day 14, the volume of the tumor was measured every 3 days using vernier calipers. On day 23, mice were euthanized by CO_2_ overdose inhalation and tumors were isolated for pathological analysis.

### Pathological tissue staining

Tumor tissues were fixed in 4% paraformaldehyde following dried and embedded in paraffin. Next, tissues were cut into 4 μm sections and deparaffinized for standard hematoxylin and eosin (HE) staining and Prussian blue staining, or deparaffinized and rehydrated for standard immunohistochemical (IHC) staining. IHC staining of ferroptosis thereof were conducted using antibodies of FTH1 (sc-376594, Santa Cruz Biotechnology) at 4 ℃ overnight, following by the second antibody (G1214, Servicebio) at 25 ℃ for 1.5 h and then stained by DAB (G1212, Servicebio) and hematoxylin. Sections were observed using an Olympus microscope (Olympus, Japan).

### Co-immunoprecipitation (co-IP) and mass spectrometry

HCC cells were lysed by Pierce™ IP Lysis Buffer (87787, Thermo Fisher Scientific) to extract protein. Protein A/G Magnetic Beads (HY-K0202, MCE) were coated with 5 μg Flag antibody at 25 ℃ for 30 min and re-suspended in 900 μL Immunoprecipitation Buffer, and 1 μg IgG antibody (sc-2025, Santa Cruz Biotechnology) was served as control. Afterwards, 100 μL cell lysis product was incubated with the 900 μL mixed magnetic beads-antibody mixture overnight at 4 °C. Finally, the bound proteins were eluted using Elution Buffer (T10007, ABmart) and subjected to mass spectrometry and WB.

### Immunofluorescence (IF) staining

To observe the co-localization of PRMT9 and HSPA8 in HepG2 cells, IF experiments were performed. Cells were fixed in 4% paraformaldehyde following permeabilized with 0.2% Triton X-100 and blocked with 3% bovine serum albumin. Next, cells were incubated with primary antibodies of PRMT9 (ab122374, abcam) and HSPA8 (66442-1-Ig, Proteintech) at 4℃ overnight, and then incubated with secondary antibody (Goat Anti-Rabbit IgG H&L (Alexa Fluor^®^ 488) (ab150077, abcam) and (H + L)(Cy3-labeled Goat Anti-Mouse IgG (H + L)) (A0521, Beyotime)) at 25 ℃ for 1 h, following by the stained with DAPI for cell nuclei at 25 ℃ for 15 min. Sections were observed using an Olympus microscope (Olympus, Japan).

### Transcriptome sequencing

HepG2 cells after overexpression HSPA8 (n = 3) was subjected to transcriptome sequencing, and HepG2 cells transfected with NC was served as control. Qualified RNA was used for cDNA library construction starting with 1 μg total RNA for each sample using the VAHTS Stranded mRNA-seq Library Prep Kit for Illumina V2 (NR612-01, Vazyme) according to the manufacturer’s instructions. Library was qualified by Agilent 2100 Bioanalyzer and sequenced on the Illumina HiSeq2500 with PE150 by Shanghai Yingbio Technology. Raw data was filtered by FastQC and mapped to human genome GRCh37. Fragments per gene were counted using STAR version 2.4.2a49 and normalized by fragments per kilo base million reads. Differentially expressed genes (DEGs) were identified by DESeq2 under the criterion: Log2FC > 1 or < − 1, p-value < 0.05. GO and KEGG analysis were conducted on DEGs.

### HCC tissue microarray

HCC tumor samples (n = 66) and adjacent normal tissues (n = 58) were collected from HCC patients who received surgical resection at the First Affiliated Hospital of Nanchang University. Written informed consent were obtained from all patients. The HCC tissue microarray was used to evaluate the expression level of PRMT9, HSPA8, and GPX4 according to the histochemistry score (H-score). For IHC staining, the progression steps were as described above. The region of interest (ROI) was segmented according to the strength of the stain and labelled accordingly using the threshold analysis module of the visiopharm software. H-Score = ∑ (pi × i) = (percentage of weak intensity × 1) + (percentage of moderate intensity × 2) + (percentage of strong intensity × 3). The pi indicates the percentage of positive signal pixel area (μm^2^)/number of cells; i represents the colouring intensity. H-score is between 0 and 300, with larger numbers indicating stronger positive intensity. 0–75 means strong positive; 76–120 means medium positive; 121–160 means weak positive; 161–212 means negative.

### Statistical analysis

The software of GraphPad Prism 9.0 (GraphPad Software, San Diego, USA) was used for graphing and statistical analysis. The data were presented as the mean ± SD of three independent experiments. The comparison between two groups was analyzed by Student’s t-test and between three or more groups was analyzed by ANOVA following Tukey test. The *p* < 0.05 was considered statistically significant.

## Results

### HBx inhibits ferroptosis and promotes PRMT9 expression in HCC

**Fig. 1:**
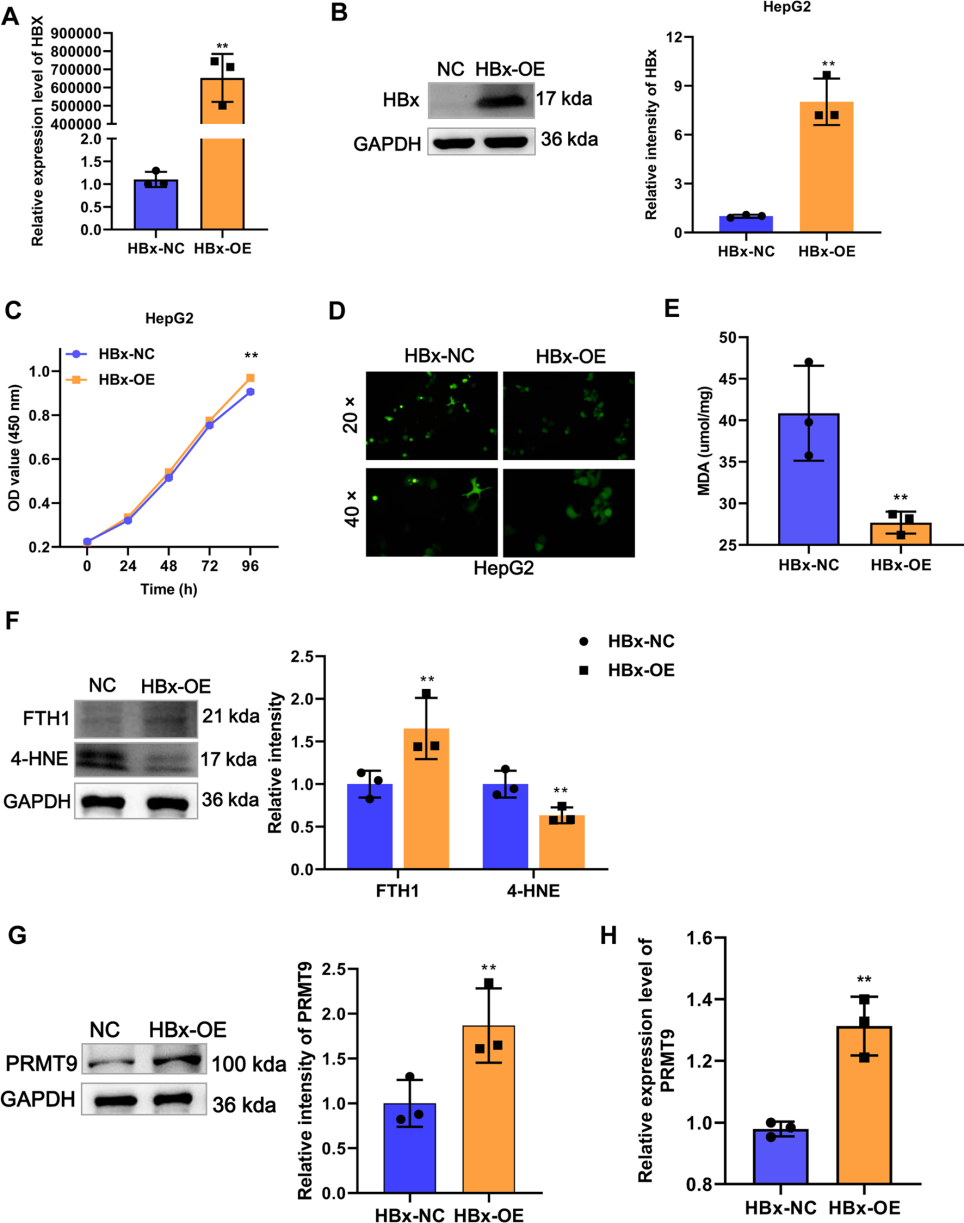
HBx inhibits ferroptosis and promotes PRMT9 expression in HCC. **A** The mRNA overexpression efficiency of HBx in HepG2 cells was detected by RT-qPCR. **B** The protein overexpression efficiency of HBx in HepG2 cells was detected by WB. **C** The proliferation of HepG2 cells was detected by CCK8 after overexpression of HBx. **D** ROS accumulation of HepG2 cells was detected by DCFH-DA kit after overexpression of HBx. **E** MDA content of HepG2 cells was detected by MDA detection kit after overexpression of HBx. **F** The ferroptosis markers 4-HNE and FTH1 expression were detected by WB after overexpression of HBx. **G** PRMT9 expression was detected by WB after overexpression of HBx. CCK-8 assays were performed in sextuplicate, and the remaining assays were triplicate. t test, **p < 0.01

To investigate the effect of HBx on ferroptosis in HCC, we first constructed an HBx overexpression plasmid using the pCDNA3.1 vector and transfected into HepG2 cells along with an empty vector as a control group. Our results demonstrated that HBx overexpression significantly enhanced the mRNA and protein expression of HBx, indicating good overexpression efficiency (Fig. 1A, B). Furthermore, we examined the effect of HBx overexpression on HepG2 cell proliferation, ROS levels, ferroptosis marker expression, and MDA content to investigate its role in ferroptosis. We found that HBx overexpression significantly promoted HepG2 cell proliferation while decreasing ROS levels and MDA content (Fig. 1C–E). Moreover, HBx overexpression significantly inhibited the ferroptosis marker 4-HNE expression while promoting the expression of FTH1 (Fig. 1F), suggesting the impairment of the ferroptosis upon HBx overexpression. In our previous work, we have reported that PRMT9 plays an important role in the progression of liver cancer. Therefore, we further investigated whether PRMT9 is involved in the regulation of ferroptosis by HBx in HCC. Compared to the control group, both the mRNA and protein expression of PRMT9 were significantly increased in the HBx overexpression group, indicating that HBx promotes the expression of PRMT9 (Fig. 1G, H). Collectively, these findings suggest that HBx inhibits ferroptosis and promotes the expression of PRMT9, thus facilitating the development of HCC.

### HBx inhibits ferroptosis in HCC cells through PRMT9

**Fig. 2:**
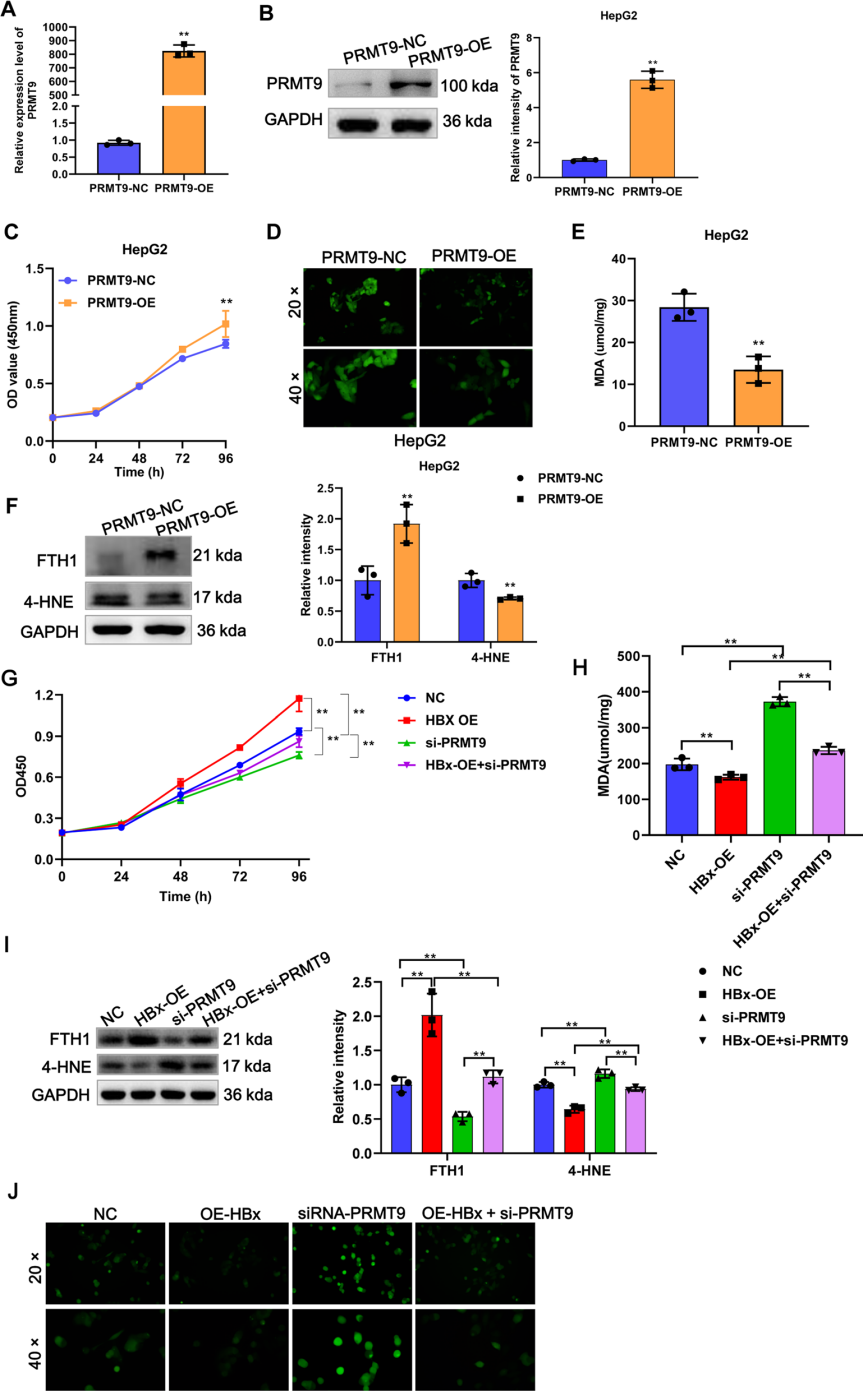
HBx inhibits ferroptosis through PRMT9 in HCC cells. The overexpression efficiency of PRMT9 at mRNA (**A**) and protein level (**B**) in HepG2 cells was detected by RT-qPCR and WB, respectively. The proliferation (**C**), ROS accumulation (**D**), MDA content (**E**), and ferroptosis markers expression (4-HNE and FTH1) (**F**) of HepG2 cells after overexpression of PRMT9 was detected by CCK8, DCFH-DA kit, MDA detection kit, and WB, respectively. **G**–**J** Rescue experiments confirmed that HBx regulated ferroptosis through PRMT9. The proliferation (**G**), MDA content (**H**), ferroptosis markers expression (4-HNE and FTH1) (**I**), and ROS accumulation (**J**) of HepG2 cells after HBx overexpression and PRMT9 knockdown was detected by CCK8, MDA detection kit, WB, and DCFH-DA kit, respectively. CCK-8 assays were performed in sextuplicate, and the remaining assays were triplicate, t test for two groups, ANOVA following Tukey’s test for four groups, **p < 0.01

To investigate the impact of PRMT9 on ferroptosis in HCC, we overexpressed and knockdown PRMT9, respectively. We previously found that PRMT9 promotes metastasis and is highly expressed in high metastatic HCC cells [12], MHCC97H is a high metastatic potential cell, and HepG2 and Huh7 are low metastatic cells, so we chose to do gain-of-function experiments in HepG2 and Huh7 cells and loss-of-function experiments in MHCC97H. As shown in Fig. 2A, B, compared with the control group transfected with empty vector, the expression of PRMT9 was significantly enhanced in the pCDNA3.1-PRMT9 overexpression group. The CCK8 results showed that compared with the control group, the proliferative capacity of HepG2 cells was significantly increased in the PRMT9 overexpression group (Fig. 2C). Further detection revealed that PRMT9 overexpression significantly reduced ROS and MDA levels in HepG2 cells compared with the control group (Fig. 2D, E). WB analysis of ferroptosis markers showed that the expression of 4-HNE was reduced but FTH1 was elevated upon PRMT9 overexpression (Fig. 2F). Subsequently, to further verify the above results, we conducted parallel experiments in Huh7 cells and obtained consistent results as described above (Additional file 1: Fig. S1A–F). The results of the PRMT9 loss-of-function also support the above conclusion, as knockdown PRMT9 in MHCC97H cells enhances ferroptosis (Additional file 1: Fig. S1G–L). In summary, these results suggest that PRMT9 suppresses ferroptosis in HCC cells.

To confirm whether the inhibitory function of HBx on ferroptosis was via PRMT9, we overexpressed HBx and knocked down PRMT9 expression simultaneously in MHCC97H cells. Compared to the control group, overexpression of HBx resulted in a significant decrease in ROS and MDA levels in cells, an increase in proliferation, and a significant expression change in FTH1 and 4-HNE (Fig. 2G–J), however, knocking down PRMT9 partially rescued the effect of HBx overexpression on ferroptosis (Fig. 2G–J). These results indicate that HBx regulates ferroptosis in HCC through PRMT9.

### *PRMT9 accelerates HCC progression *in vivo* through ferroptosis*

**Fig. 3:**
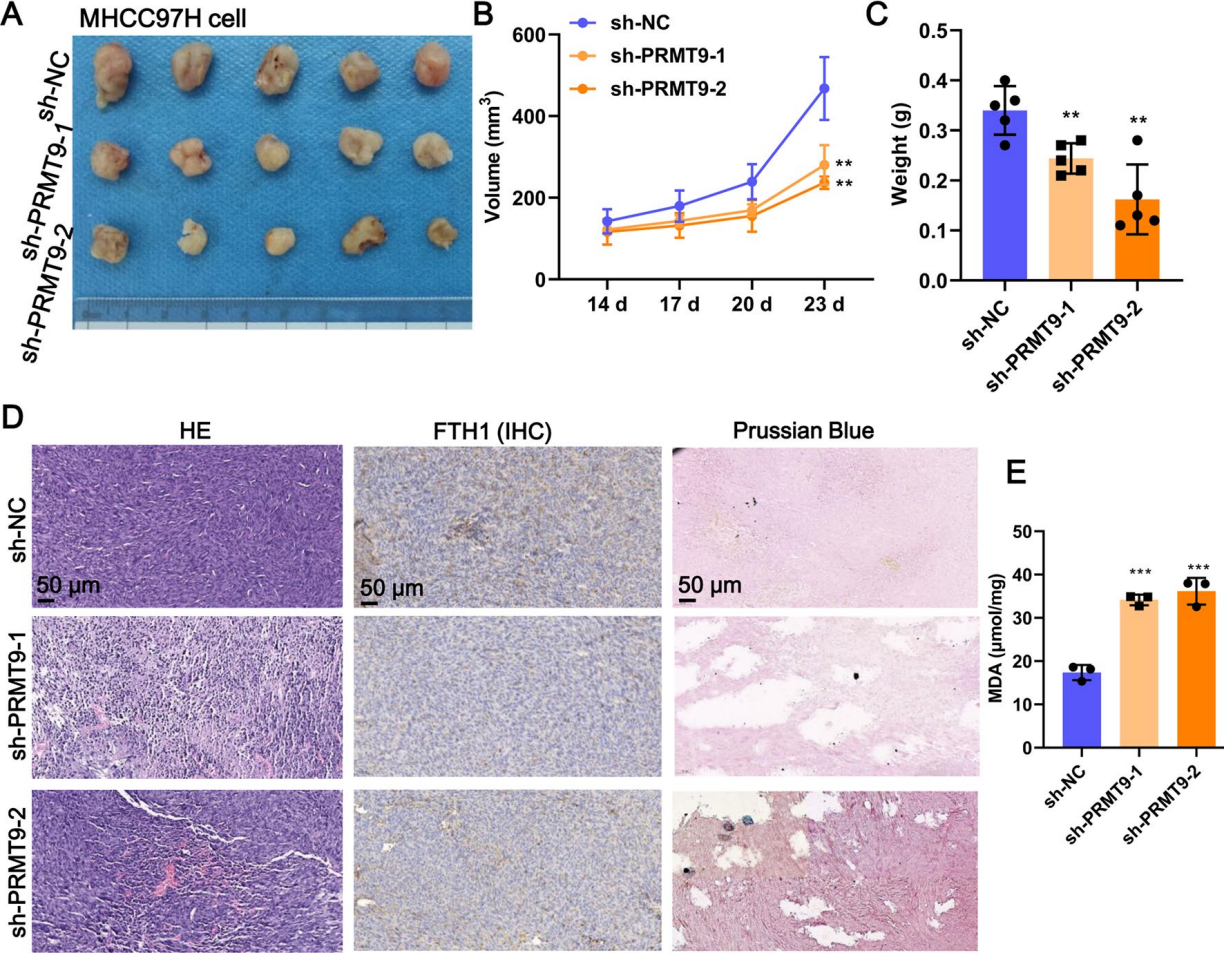
PRMT9 inhibits ferroptosis to suppress tumor growth in vivo. **A** Representative ex vivo images of tumor tissues. n = 5. **B** Tumor volume growth curves. n = 5. **C** Dissected tumor weights. n = 5. **D** Representative images of HE staining, IHC staining of FTH1 antibody, Prussian blue staining. n = 3. **E** MDA content in tumor tissues, n = 3. **p < 0.01, *** *p* < 0.001

To further verify PRMT9 suppresses ferroptosis in HCC in vivo, the xenograft tumor mouse model using PRMT9-deleted MHCC97H cells was established (Fig. 3A). The results showed that tumor volume and weight were significantly diminished in nude mice in response to PRMT9 knockdown (Fig. 3B, C). HE staining reveled histological abnormalities in tumor tissues (Fig. 3D). Moreover, Prussian Blue staining revealed a heavier accumulation of iron in HCC tumor tissues after PRMT9 knockdown (Fig. 3D). PRMT9 knockdown also significantly suppressed FTH1 protein expression (Fig. 3D) and enhanced MDA content (Fig. 3E) in HCC tumor tissues. In contrast, overexpression of PRMT9 significantly promoted tumor growth and inhibited the ferroptosis flux, as evidenced by the Additional file 2: Fig. S2. Taken together, PRMT9 inhibits ferroptosis to accelerate HCC development in vivo.

### PRMT9 enhances arginine methylation of HSPA8 in HCC

**Fig. 4:**
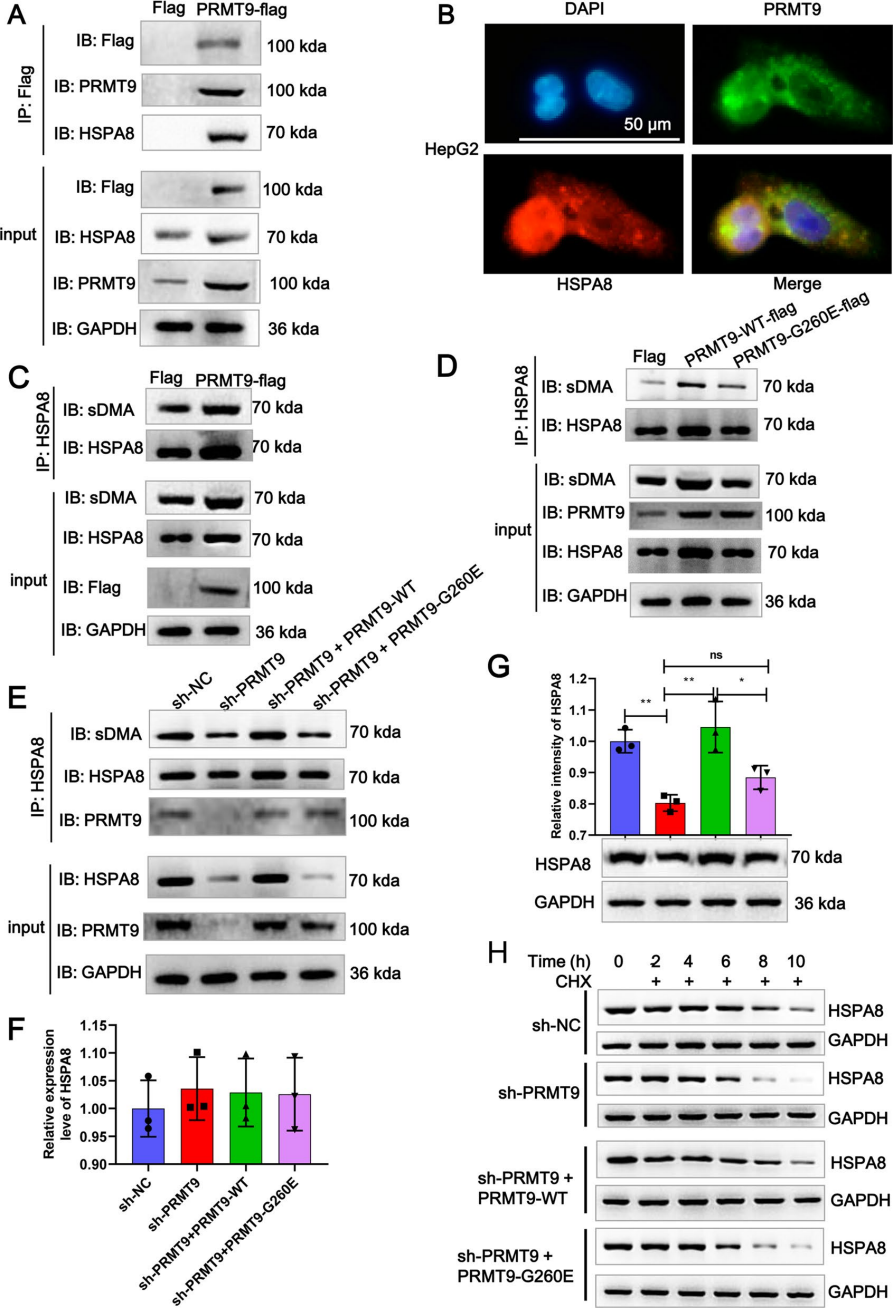
PRMT9 enhances arginine methylation of HSPA8 in HCC. **A** PRMT9-flag and HSPA8 co-immunoprecipitated in HepG2 cells after transfecting with PRMT9-flag or flag. Total cell lysates were incubated with flag and the eluted protein was detected by WB using antibodies of flag, PRMT9, HSPA8, GAPDH. **B** The colocalization between PRMT9 and HSPA8 was assessed by IF assay in HepG2 cells. **C** PRMT9-flag and sDMA on HSPA8 co-immunoprecipitated in HepG2 cells after transfecting with PRMT9-flag or flag. Total cell lysates was incubated with HSPA8 antibody and the eluted protein was detected by WB using antibodies of sDMA, flag, HSPA8, GAPDH. **D** Flag, PRMT9-WT-flag or PRMT9 mutant G260E (PRMT9-G260E-flag) were transfected together with HSPA8 into HepG2 cells. Total cell lysates was incubated with HSPA8 antibody and the eluted protein was detected by WB using antibodies of sDMA, PRMT9, HSPA8, GAPDH. **E** Transfected PRMT9-WT-flag or PRMT9-G260E-flag into PRMT9-deleted HepG2 cells, and then total cell lysates was incubated with HSPA8 antibody and the eluted protein was detected by WB using antibodies of sDMA, PRMT9, HSPA8, GAPDH. Transfected PRMT9-WT-flag or PRMT9-G260E-flag into PRMT9-deleted HepG2 cells, and then the HSPA8 mRNA expression was detected by RT-qPCR (**F**), the HSPA8 mRNA expression was detected by WB (**G**). **H** Transfected PRMT9-WT-flag or PRMT9-G260E-flag into PRMT9-deleted HepG2 cells, and treated with CHX. The HSPA8 protein expression was detected at different time point using WB. ANOVA following Tukey’s test, n = 3, ns *p* > 0.05, * *p* < 0.05, **p < 0.01

PRMT9 mainly regulates the formation of monomethylarginine (MMA) and symmetric dimethylarginine (sDMA) [13]. To investigate whether PRMT9 inhibits ferroptosis in HCC cells by regulating arginine methylation, we first used WB to detect the overall levels of MMA and sDMA in Huh7 cells upon overexpressing PRMT9. The results showed that the levels of MMA and sDMA in Huh7 cells were significantly increased after overexpression of PRMT9 (Additional file 3: Fig. S3A). Subsequently, to study target proteins modified by PRMT9, we transfected Huh7 cells with a flag-tagged PRMT9 and flag antibody was used for IP (Additional file 3: Fig. S3B). In addition, to detect proteins that underwent arginine methylation, we used MMA and sDMA antibodies for IP experiments (Additional file 3: Fig. S3B). Due to the more significant impact of PRMT9 on sDMA and the more pronounced sDMA specific bands in IP results, subsequent IP experiments only studied sDMA. Mass spectrometry analysis was performed on specific bands to obtain target proteins. A total of 148 and 266 proteins were identified in the PRMT9-flag and sDMA groups, respectively, among which 61 proteins were shared between the two groups. These proteins may undergo arginine methylation modification mediated by PRMT9 (Additional file 3: Fig. S3C). Further analysis of the shared 61 proteins revealed that there were a large number of tumor-related proteins, such as HSPA5, HNRNPH1, HSPA8, TGM3, and SERPINB12 (Additional file 3: Fig. S3C). Among them, HSPA8 is associated with ferroptosis [14] and can be regulated by HBx [15], hence we focus on HSPA8. A flag-tagged monoclonal antibody, which specifically recognize the flag-tagged PRMT9, was generated to verify binding to HSPA8. We found that flag-tagged PRMT9 and HSPA8 were reciprocally co-immunoprecipitated from HepG2 cells (Fig. 4A). Strikingly, a clear co-localization of PRMT9 and HSPA8 in HepG2 cells (Fig. 4B) and MHCC97H cells (Additional file 3: Fig. S3D) were observed. Furthermore, co-IP experiments with HSPA8 antibody were performed, and WB confirmed that the sDMA enrichment in HSPA8 antibody was elevated in the HepG2 cells after transfected PRMT9-flag overexpression vector compared with flag vector (Fig. 4C). Therefore, these results indicated that PRMT9 regulated the arginine methylation of HSPA8.

Mutation of Gly260 renders PRMT9 into a catalytically inactive form [16]. To investigate whether PRMT9-mediated HSPA8 arginine methylation depends on its methyltransferase activity, PRMT9-WT-flag or PRMT9 mutant G260E (PRMT9-G260E-flag) were transfected together with HSPA8 into HepG2 cells. As shown in Fig. 4D, the PRMT9-WT-flag increased the sDMA abundance of HSPA8, and the G260E mutation resulted in the loss of this ability. We also reintroduced PRMT9-WT-flag and PRMT9-G260E-flag into PRMT9-deleted HepG2 cells and detected HSPA8 methylation. The results indicated that the sDMA abundance of HSPA8 in PRMT9-deleted HepG2 cells was restored by PRMT9-WT-flag, whereas the PRMT9-G260E-flag had no such effect (Fig. 4E). Overall, these data implicated that PRMT9 catalyzed HSPA8 methylation via the methyltransferase activity.

To investigate whether PRMT9-mediated arginine methylation of HSPA8 affects its expression, we first examined mRNA expression which showed no change (Fig. 4F). WB revealed that PRMT9 knockdown significantly inhibited HSPA8 expression and that the PRMT9-WT overexpression plasmid restored its expression, but the PRMT9-G260E mutant plasmid did not have this effect (Fig. 4G). It has been shown that PRMT9 can affect the protein stability of the target, so we tested whether PRMT9 regulates HSPA8 protein levels through stability. CHX assay results showed that PRMT9 methyl transfer activity affects HSPA8 expression and protein stability (Fig. 4H). These data indicated that PRMT9 relies on arginine activity to enhance HSPA8 protein activity.

### PRMT9 inhibits ferroptosis through arginine methyltransferase activity in HCC

**Fig. 5:**
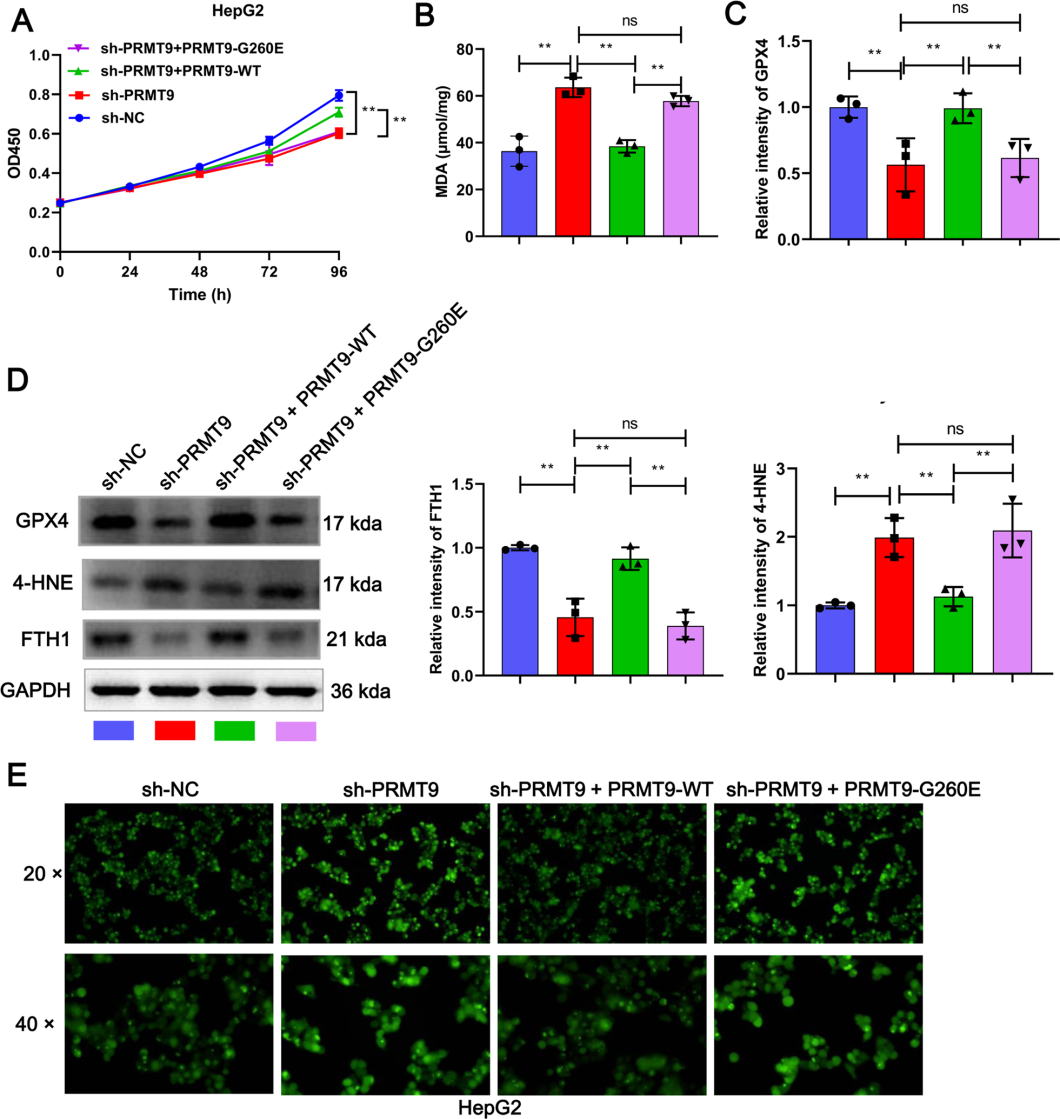
PRMT9 inhibits ferroptosis through arginine methylation of HSPA8 in HCC. Transfected PRMT9-WT-flag or PRMT9-G260E-flag into PRMT9-deleted HepG2 cells, and then the proliferation was detected by CCK8 (**A**), MDA content was detected by  the MDA detection kit (**B**), the expression of GPX4, 4-HNE, and FTH1 were detected by WB (**C**, **D**), ROS accumulation was detected by the DCFH-DA kit (**E**). CCK-8 assays were performed in sextuplicate, and the remaining assays were triplicate, ANOVA following Tukey’s test, ns *p* > 0.05, **p < 0.01

To further determine whether PRMT9-mediated ferroptosis suppression depends on HSPA8 arginine methylation, the ferroptosis level of PRMT9-deleted HepG2 cells was assessed in the presence of PRMT9-WT-flag or PRMT9-G260E-flag. As expected, the expansion of ferroptosis flux induced by sh-PRMT9 was counteracted by PRMT9-WT-flag, however this effect was lost after G260E mutation, as evidenced by decreased cell proliferation, increased MDA and ROS level, down-regulated GPX4 and FTH1 expression, and up-regulated 4-HNE expression in the G260E mutant group compared to the PRMT9-WT-flag group (Fig. 5). Therefore, PRMT9 inhibits ferroptosis in HCC by regulating arginine methyltransferase activity.

### PRMT9 inhibits ferroptosis through arginine methylation of HSPA8 at R76 and R100

**Fig. 6:**
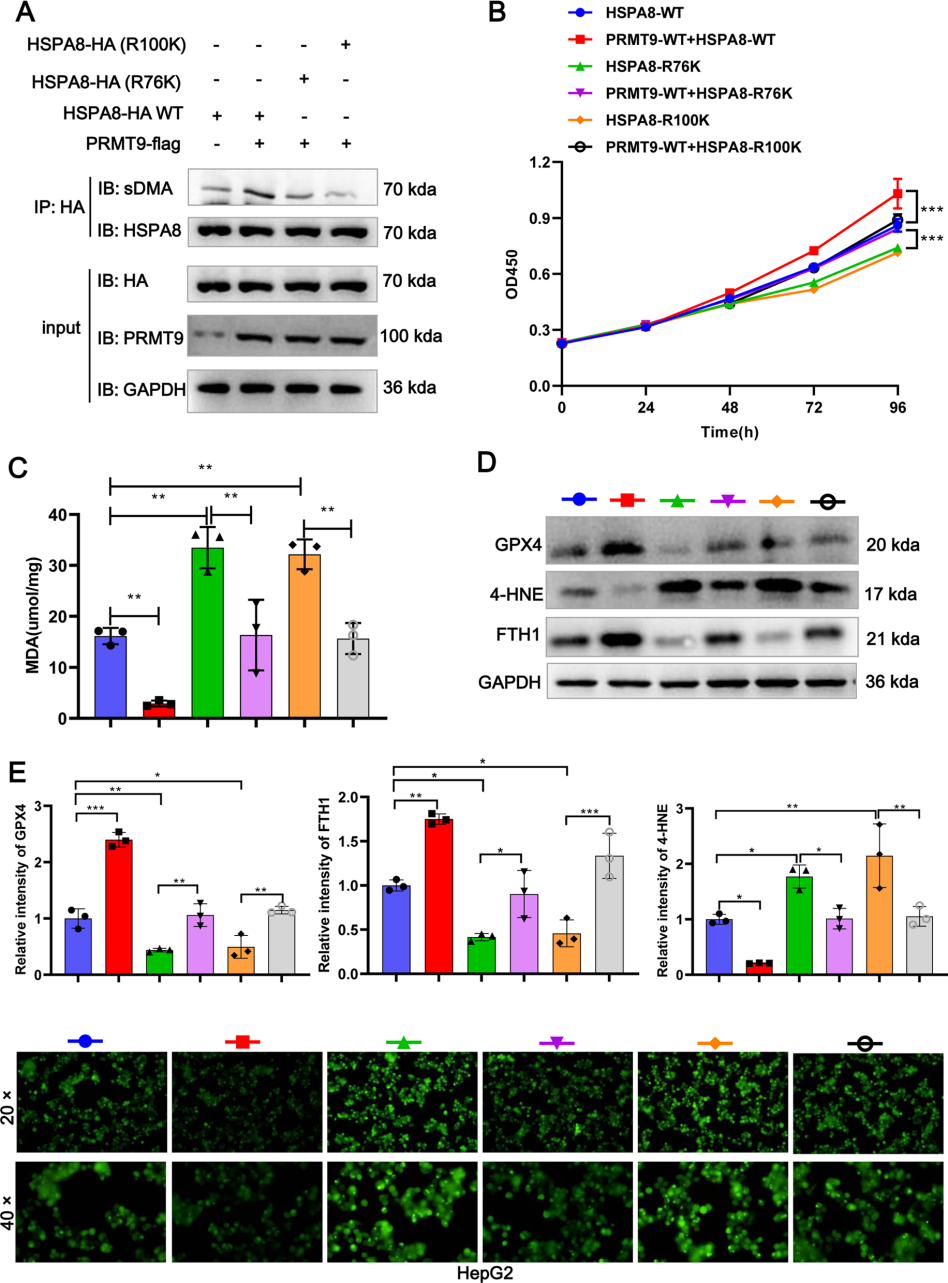
PRMT9 inhibits ferroptosis through arginine methylation of HSPA8 at R76 and R100. Mutate arginine (R76 and R100) to lysine (R76K and R100K) on HSPA8, and PRMT9-WT-flag overexpression vector was co-transfected with HA-HSPA8 WT, HA-HSPA8 (R76K), or HA-HSPA8 (R100K) into HSPA8-deificiency HepG2 cells, and then CoIP experiments were performed using HA antibody, and sDMA expression was detected by WB (**A**), the proliferation was detected by CCK8 (**B**), MDA content was detected by MDA detection kit (**C**), the expression of HSPA8, GPX4, 4-HNE, and FTH1 were detected by WB (**D**), grayscale statistical results of the blot (**E**), ROS accumulation was detected by DCFH-DA kit (**F**). CCK-8 assays were performed in sextuplicate, and the remaining assays were triplicate. ANOVA following Tukey’s test, ns *p* > 0.05,*p < 0.05, **p < 0.01, ***p < 0.001

To explore the potential arginine residues on HSPA8 that are modified by PRMT9, we predicted the sDMA modification site of HSPA8 using the Protein Methylation Prediction Database (http://msp.biocuckoo.org/index.php), as shown in the Additional file 4: Fig. S4A, B. We selected the two highest scoring arginine modification sites in the HSPA8 sequence for mutation validation experiments to mutate arginine (R76 and R100) to lysine (R76K and R100K) (Additional file 4: Fig. S4C). The PRMT9-WT-flag overexpression vector was transfected into HSPA8 deficiency HepG2 cells, followed by HA-HSPA8 WT, HA-HSPA8 (R76K), and HA-HSPA8 (R100K) overexpression vectors, respectively. Subsequently, CoIP experiments were performed using HA antibody, and sDMA level was detected by WB. The results showed that PRMT9-flag increase the methylation of HSPA8 (WT), whereas mutations at both the R76 and R100 sites resulted in a reduction enrichment of sDMA on HSPA8 in the presence of PRMT9 (Fig. 6A). Taken together, these results suggested that PRMT9 catalyzes HSPA8 arginine methylation at R76 and R100.

To further determine whether the PRMT9 dependents on HSPA8 arginine methylation to regulate ferroptosis, we introduced PRMT9-WT-flag, HSPA8 WT, HSPA8-R76K, and HSPA8-R100K overexpression plasmids into HSPA8 deficiency HepG2 cells, respectively. The results demonstrated that, compared with the HSPA8 WT group, the ferroptosis level was significantly enhanced in the HSPA8-R76K and HSPA8-R100K group (Fig. 6B–F). Similarly, PRMT9 significantly inhibited ferroptosis in HCC cells in the presence of HSPA8 WT, whereas the inhibition effect of PRMT9 on ferroptosis was diminished after mutation at R76 and R100 on HSPA8 (Fig. 6B–F). Therefore, PRMT9 inhibits ferroptosis through arginine methylation of HSPA8 at R76 and R100.

### HSPA8 overexpression alters the transcriptome profile of HCC cells

**Fig. 7:**
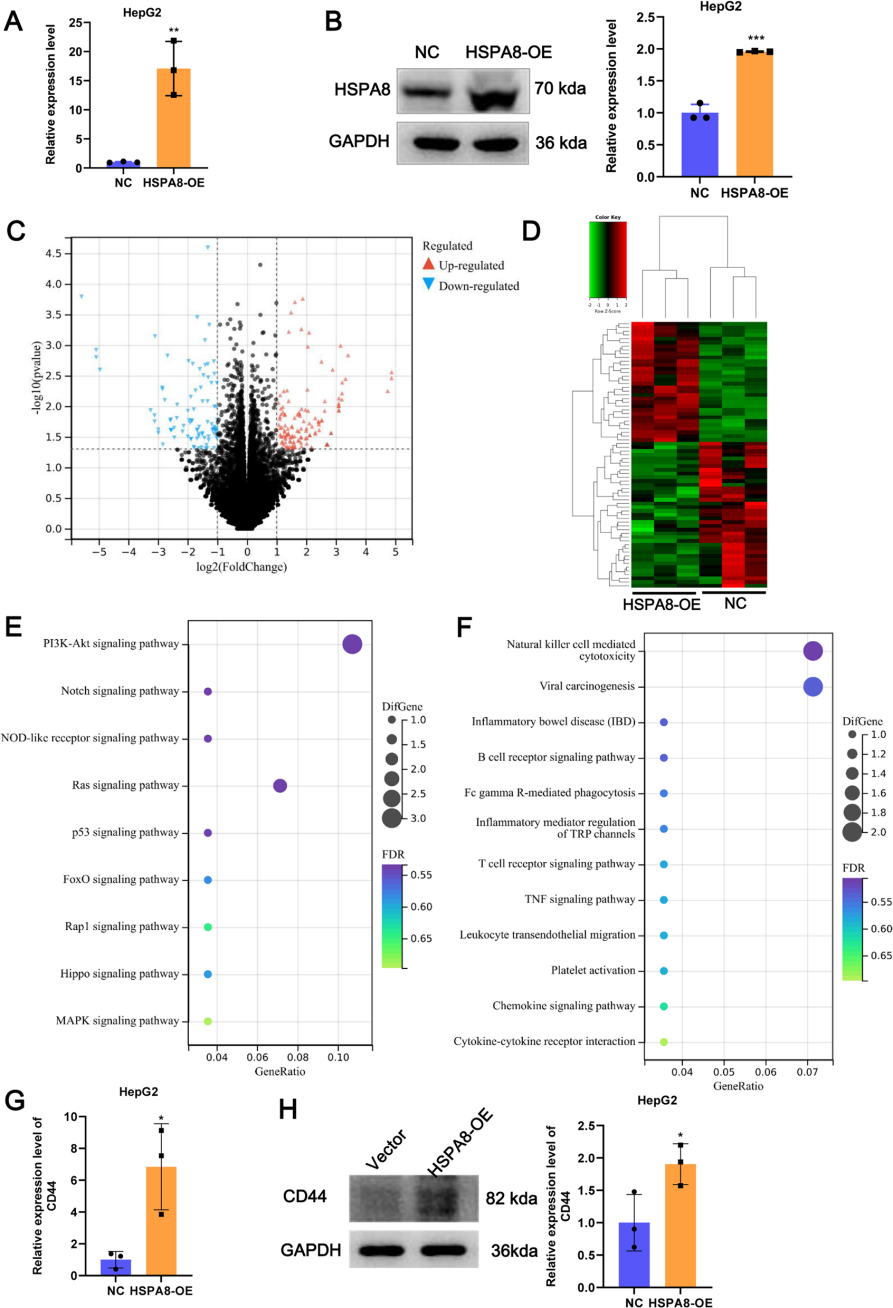
HSPA8 overexpression alters the transcriptome profile of HepG2 cells. The overexpression efficiency of HSPA8 at mRNA (**A**) and protein level (**B**) in HepG2 cells was detected by RT-qPCR and WB, respectively. (**C**) Volcano map of the DEGs. Red indicates DEGs that are up-regulated in the HSPA8 overexpression group and blue indicates DEGs that are down-regulated in the HSPA8 overexpression group. **D** Clustering heatmap of DEGs. **E** DEG-enriched ferroptosis-associated KEGG pathway bubble map. **F** DEG-enriched immune-associated KEGG pathway bubble map. **G** The mRNA expression of CD44 in HepG2 cells was detected by RT-qPCR. **H** The protein expression of CD44 in HepG2 cells was detected by WB. All assays were triplicate, t test, *p < 0.05, **p < 0.01, ***p < 0.001

To screen for key genes downstream of HSPA8, HepG2 cells overexpressing HSPA8 (Fig. 7A, B) were used for transcriptome sequencing, with a transfected blank vector as a control group. A total of 221 DEGs were identified, of which 131 were up-regulated (including SOX10 and CD44) and 90 were down-regulated in the HSPA8 overexpression group compared to the control group (Fig. 7C). The clustering heat map showed a significantly different expression pattern of DEGs between the two groups (Fig. 7D). KEGG enrichment bubble plots revealed that HSPA8 overexpression-induced DEGs were enriched in ferroptosis-related pathways, such as p53 signaling pathway and Notch signaling pathway (Fig. 7E). Interestingly, we also found that many immune-related signaling pathways were also enriched by these DEGs, including natural killer cell mediated cytotoxicity, B cell receptor signaling pathway, and T cell receptor signaling pathway (Fig. 7F), suggesting that the transcriptome alterations caused by HSPA8 overexpression may be related to the immune regulation. In summary, HSPA8 overexpression alters the transcriptome profile of HepG2 cells.

### *PRMT9/HSPA8 regulates ferroptosis *via* CD44*

**Fig. 8:**
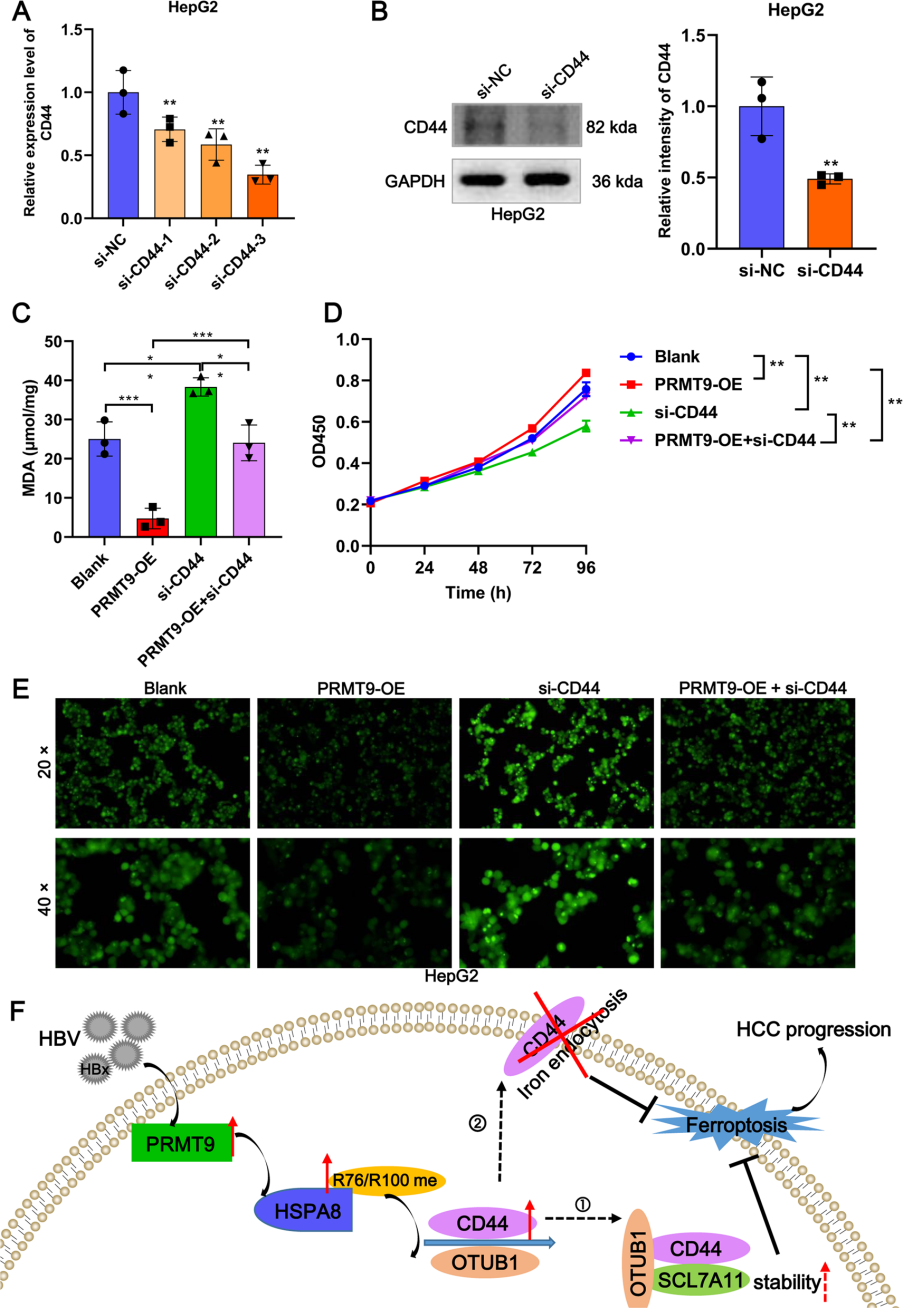
PRMT9/HSPA8 regulates ferroptosis via CD44. The knockdown efficiency of CD44 at mRNA (**A**) and protein level (**B**) in HepG2 cells was detected by RT-qPCR and WB, respectively. After co-transfection of PRMT9 overexpression vector and siRNA targeting CD44, MDA content was detected by MDA detection kit (**C**), cell proliferation proliferation was detected by CCK8 (**D**), ROS accumulation was detected by DCFH-DA kit (**E**). **F** Diagram of the hypothesis of the HBx/PRMT9/HSPA8/CD44 axis in the regulation of ferroptosis in an arginine methylation-dependent manner and progression of HCC. The solid line represents the results of this study, while the dashed line of ① and ② represents previous research. ①: CD44 regulates intracellular free iron ion content through the endocytosis of iron-bound hyaluronates [32]. ② CD44 inhibits ferroptosis by enhancing the stability of SLC7A11 in an OTUB1-dependent manner [34]. CCK-8 assays were performed in sextuplicate, and the remaining assays were triplicate, t test for two groups, ANOVA following Tukey’s test for three and four groups, *p < 0.05, **p < 0.01, ***p < 0.001

A literature search preliminarily explored the relationship between 221 DEGs and ferroptosis, among which only SOX10 and CD44 inhibit ferroptosis [17, 18]. So they were initially targeted, but as only CD44 was included in the ferroptosis database FerrDb V2 (http://www.zhounan.org/ferrdb/current/), CD44 was ultimately selected as the downstream target gene for HSPA8. Up-regulated expression of CD44 in HepG2 cells upon HSPA8 overexpression was confirmed by RT-qPCR (Fig. 7G) and WB (Fig. 7H). To investigate whether the PRMT9/HSPA8 axis regulates ferroptosis in HCC cells via CD44, we transfected siNRAs targeting CD44 into PRMT9 overexpressing HepG2 cells. The si-CD44-3 significantly inhibited the expression of endogenous CD44 in HepG2 cells (Fig. 8A, B). Moreover, si-CD44 significantly promoted ferroptosis flux and reversed the inhibition of ferroptosis caused by PRMT9 overexpression, as indicated by the changes in proliferation, MDA, ROS, and FTH1 expression (Fig. 8C–E). In all, PRMT9/HSPA8 regulates ferroptosis via CD44 to involve in HCC.

### PRMT9 and HSPA8 expression were prognostic marker for HCC

**Fig. 9:**
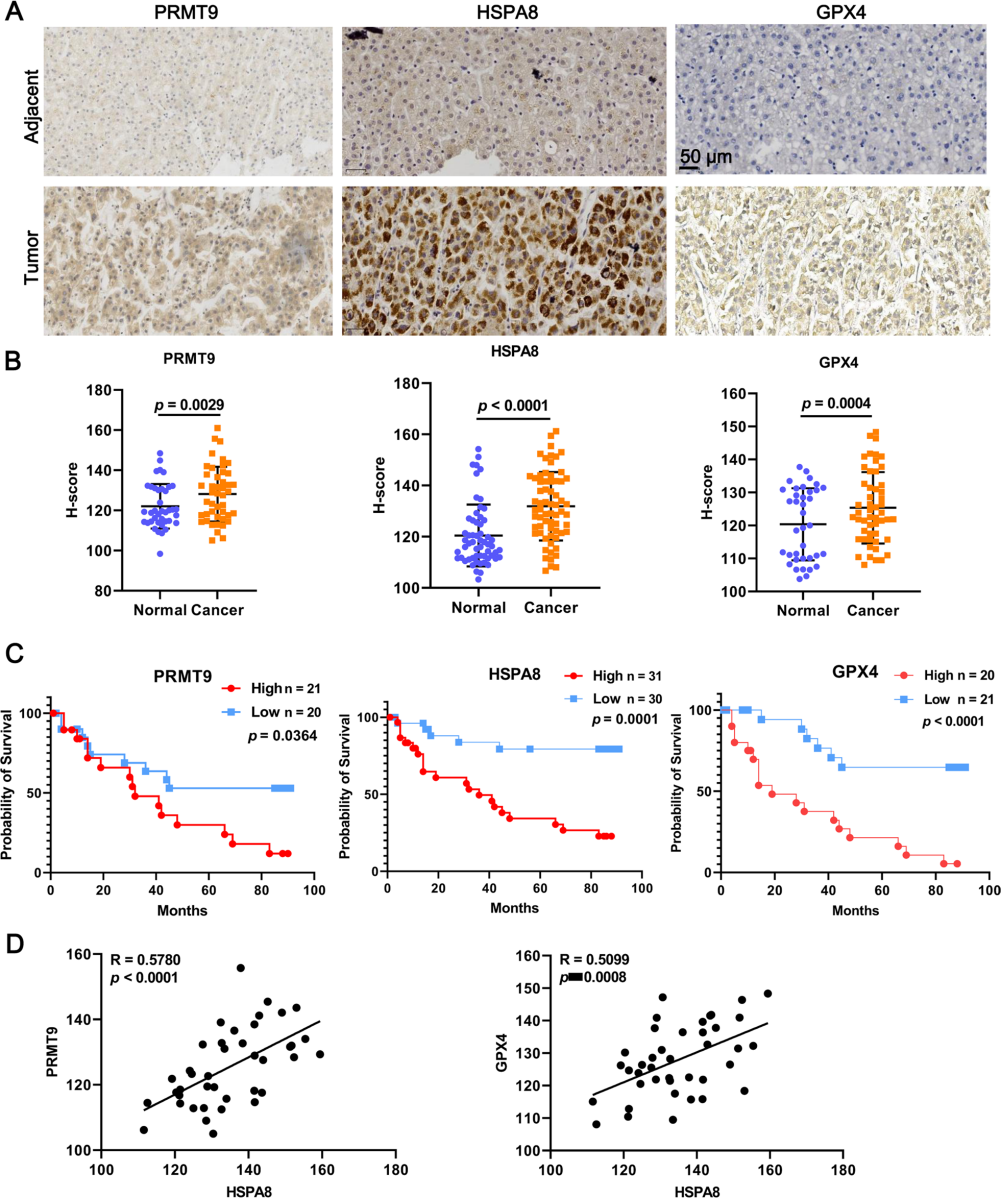
HSPA8 expression was a prognostic marker for HCC. **A** IHC for PRMT9, HSPA8, and GPX4 was performed in human HCC tissue microarray. **B** H-scores were calculated to characterise gene expression levels. PRMT9, n = 45 for tumor tissues and n = 40 for normal tissues. HSPA8, n = 66 for tumor tissues and n = 58 for normal tissues. GPX4, n = 52 for tumor tissues and n = 38 for normal tissues. **C** Kaplan–Meier survival analysis comparing overall survival between patients with high and low expression levels of PRMT9 (n = 41), HSPA8 (n = 61), and GPX4 (n = 41) based on H-score. **D** Correlation analysis of final H-scores between PRMT9, HSPA8, and GPX4. n = 40

Finally, a tissue microarray including 66 HCC samples and 58 normal tissues was used for validation by IHC. The results showed that the expression of PRMT9, HSPA8, and GPX4 were significantly higher in tumor than that in adjacent tissues (Fig. 9A, B). Interestingly, higher expression of PRMT9, HSPA8, and GPX4 based on H-score were indicated poor overall survival time (Fig. 9C). Microarray results showed HSPA8 expression was positively correlated with PRMT9 (R = 0.5780, *p* < 0.0001) and GPX4 (R = 0.5099, *p* = 0.0008) (Fig. 9D). These data implicated that the expression of PRMT9 and HSPA8 could be served as prognostic marker for HCC.

## Discussion

HBx is independently associated with HBV-related HCC progression, and how HBx proteins induce HCC progression has been the key question that needs to be addressed urgently. The deepening understanding of ferroptosis has brought dawn to the molecular mechanism of HBx-induced HCC. This study represents the first discovery that PRMT9-mediated arginine methylation of HSPA8 is a key signal regulating ferroptosis in HCC cells, and blocking the methylation modification of HSPA8 by knocking down PRMT9 significantly promoted ferroptosis through CD44 in HCC cells leading to impaired HCC progression (Fig. 8F).

PRMT9, also known as FBXO11, was identified in 2006 by Cook et al. [11]. PRMT9 is the molecule in the family of PRMTs responsible for the formation of sDMA modifications in proteins. Although numerous studies suggesting the involvement of FBXO11 in tumor progression [12, 19, 20], PRMT9-mediated arginine methylation modifications in tumors have not been studied much, limited by the short time frame of the studies. Only 20 articles related to “PRMT9” were retrieved from the PubMed database, while only 4 articles were retrieved under the keyword "PRMT9 SDMA", and none of them were related to the functional studies of PRMT9 in tumors. Therefore, in the present study, PRMT9 methylation modifies HSPA8 to regulate HCC progression, to some extent bridging the gap in the field. Moreover, the study of FBXO11/PRMT9 in ferroptosis has also not been reported. In short, this study is the first to demonstrate that PRMT9-mediated arginine methylation modifications regulate ferroptosis and thus participate in HBx-induced HCC progression.

In this study, we found that the regulation of ferroptosis by PRMT9 is dependent on arginine methylation of HSPA8 at R76 and R100. HSPA8 is a chaperone protein that plays a role in a variety of cellular biological processes [21], such as ferroptosis [14], autophagy [22], and cell senescence [23]. In addition, HSPA8 is a member of the HSP family, which has been proposed as an endogenous modulators of ferroptosis as a double role [24]. For example, HBx-induced up-regulation of HSPA8 inhibits ferroptosis to support HCC progression [25]. Suppression of HSPA8 by rifampicin promotes ferroptosis in anti-tuberculosis drug-induced liver injury [14]. The formation of HSPA8-LAMP2A-GPX4 protein complex promotes GPXA degradation and thus facilitate ferroptosis [26, 27]. These studies suggest that HSPA8 is a regulator of ferroptosis. Moreover, in this study, we found that the regulation of ferroptosis by HSPA8 was dependent on the arginine methylation modification of its R76 and R100 site, and that mutation of R76 and R100 significantly reversed the inhibition of ferroptosis caused by overexpression of PRMT9. However, no further HSPA8 arginine methylation modifications have been reported, except for the one, HSPA8 highly methylated by PRMT7 at site of FELTGIPPAPR-469 in HCT116 cells, reported by Magdalena et al. [28]. Therefore, this study is the first to reveal the molecular mechanism by which PRMT9 methylated HSPA8 to regulate ferroptosis in HCC cells. These data provide a valuable resource for future studies of HBx-induced HCC from the perspective of arginine methylation/HSAP8/ferroptosis.

In addition, we also identified that CD44 is a downstream target gene for HSPA8 to regulate ferroptosis to involve in HCC progression. CD44 is a single-transmembrane glycoprotein and its role in cancer development has been widely reported [29–31]. There are two possible pathways for CD44-mediated ferroptosis. One is that CD44 regulates intracellular free iron ion content. Sebastian et al. proved that CD44 could mediate the endocytosis of iron-bound hyaluronates to control epigenetic plasticity in tumor cells [32]. Iron oxide nanoparticles can increase labile iron pool-induced ferroptosis in chemoresistant breast cancer cells for therapeutic purposes through CD44-mediated endocytosis [33]. The second is that CD44 inhibits ferroptosis by enhancing the stability of SLC7A11 in an OTUB1-dependent manner [34]. Of course, given the limited nature of current knowledge, it is likely that one or more unknown pathways exist between CD44 and ferroptosis. In all, these studies support our conclusion that CD44 regulates ferroptosis in HCC cells.

## Conclusion

In summary, this study demonstrated for the first time that HBx/PRMT9/HSPA8/CD44 axis controlled ferroptosis in an arginine methylation-dependent manner in HCC cells. The results of this study not only contribute to the in-depth study of the mechanism of HBV-induced HCC, but also provide promising small molecule strategies for targeted treatment of HCC.

### Supplementary Information

**Additional file 1: Figure S1. **PRMT9 regulates ferroptosis in HCC cells. The overexpression efficiency of PRMT9 at mRNA (A) and protein level (B) in Hhu7 cells was detected by RT-qPCR and WB, respectively. The proliferation (C), ROS accumulation (D), MDA content (E), and ferroptosis markers expression (4-HNE and FTH1) (F) of Hhu7 cells after overexpression of PRMT9 was detected by CCK8, DCFH-DA kit, MDA detection kit, and WB, respectively. The knockdown efficiency of PRMT9 at mRNA (G) and protein level (H) in MHCC97H cells was detected by RT-qPCR and WB, respectively. The proliferation (I), MDA content (J), ROS accumulation (K), and ferroptosis markers expression (4-HNE and FTH1) (L) of MHCC97H cells after knockdown of PRMT9 was detected by CCK8, MDA detection kit, DCFH-DA kit, and WB, respectively. CCK-8 assays were performed in sextuplicate, and the remaining assays were triplicate, t test for two groups, ANOVA following Tukey’s test for three and four groups, ns *p* > 0.05,* *p* < 0.05, ** *p* < 0.01, *** *p* < 0.01

**Additional file 2: Figure S2. **Overexpression of PRMT9 promotes HCC in mice. (A) Representative ex vivo images of tumor tissues. n = 5. (B) Tumor volume growth curves. n = 5. (C) Dissected tumor weights. n = 5. (D) Representative images of HE staining, IHC staining of FTH1 antibody, Prussian blue staining. n = 3. (E) MDA content in tumor tissues. n = 3. ** *p* < 0.01, *** *p* < 0.001

**Additional file 3: Figure S3. **PRMT9 targets HSPA8 in HCC cells. (A) The protein expression of MMA and sDMA in Hhu7 cells after overexpression of PRMT9 was detected by WB. n = 3, t test, * *p* < 0.05, ** *p* < 0.01. (B) Flag-tag purification of the PRMT9 protein complex from Hhu7 cells, MMA co-immunoprecipitated protein complex, and sDMA co-immunoprecipitated protein complex, these eluted protein complex was separated by SDS–PAGE and silver-stained, and then identified by mass spectrometry. (C) A total of 61 proteins was overlapped by immunoprecipitation of PRMT9-flag and sDMA. (D) The colocalization between PRMT9 and HSPA8 was assessed by IF assay in MHCC97H cells.

**Additional file 4: Figure S4. **The potential arginine residues on HSPA8 that are modified by PRMT9. The predicted sDMA modification site (A) and predicted score (B) of HSPA8 using the Protein Methylation Prediction Database. (C) Amino acid sequence of HSPA8 protein. The yellow background marks the sequence for the validation site, mutating R76 and R100 to R76K and R100K, respectively.

**Additional file 5: Table S1**. The primers used in this study.

## Data Availability

All data used in this work can be acquired from the corresponding author upon reasonable request.

## References

[1] El-Serag HB (2012). Epidemiology of viral hepatitis and hepatocellular carcinoma. Gastroenterology.

[2] Zhang XD, Wang Y, Ye LH (2014). Hepatitis B virus X protein accelerates the development of hepatoma. Cancer Biol Med.

[3] Keng VW, Tschida BR, Bell JB, Largaespada DA (2011). Modeling hepatitis B virus X-induced hepatocellular carcinoma in mice with the Sleeping Beauty transposon system. Hepatology.

[4] Tang D, Chen X, Kang R, Kroemer G (2021). Ferroptosis: molecular mechanisms and health implications. Cell Res.

[5] Yao F, Deng Y, Zhao Y, Mei Y, Zhang Y, Liu X (2021). A targetable LIFR-NF-kappaB-LCN2 axis controls liver tumorigenesis and vulnerability to ferroptosis. Nat Commun.

[6] Liu GZ, Xu XW, Tao SH, Gao MJ, Hou ZH (2021). HBx facilitates ferroptosis in acute liver failure via EZH2 mediated SLC7A11 suppression. J Biomed Sci.

[7] Kuo CY, Chiu V, Hsieh PC, Huang CY, Huang SJ, Tzeng IS (2020). Chrysophanol attenuates hepatitis B virus X protein-induced hepatic stellate cell fibrosis by regulating endoplasmic reticulum stress and ferroptosis. J Pharmacol Sci.

[8] Yang Y, Bedford MT (2013). Protein arginine methyltransferases and cancer. Nat Rev Cancer.

[9] Wang YP, Zhou W, Wang J, Huang X, Zuo Y, Wang TS (2016). Arginine methylation of MDH1 by CARM1 inhibits glutamine metabolism and suppresses pancreatic cancer. Mol Cell.

[10] Liu L, Zhang X, Ding H, Liu X, Cao D, Liu Y (2021). Arginine and lysine methylation of MRPS23 promotes breast cancer metastasis through regulating OXPHOS. Oncogene.

[11] Cook JR, Lee JH, Yang ZH, Krause CD, Herth N, Hoffmann R (2006). FBXO11/PRMT9, a new protein arginine methyltransferase, symmetrically dimethylates arginine residues. Biochem Biophys Res Commun.

[12] Jiang H, Zhou Z, Jin S, Xu K, Zhang H, Xu J (2018). PRMT9 promotes hepatocellular carcinoma invasion and metastasis via activating PI3K/Akt/GSK-3beta/Snail signaling. Cancer Sci.

[13] Yang Y, Hadjikyriacou A, Xia Z, Gayatri S, Kim D, Zurita-Lopez C (2015). PRMT9 is a type II methyltransferase that methylates the splicing factor SAP145. Nat Commun.

[14] Zhou J, Tan Y, Hu L, Fu J, Li D, Chen J (2022). Inhibition of HSPA8 by rifampicin contributes to ferroptosis via enhancing autophagy. Liver Int.

[15] Wang YP, Liu F, He HW, Han YX, Peng ZG, Li BW (2010). Heat stress cognate 70 host protein as a potential drug target against drug resistance in hepatitis B virus. Antimicrob Agents Chemother.

[16] Bai X, Sui C, Liu F, Chen T, Zhang L, Zheng Y (2022). The protein arginine methyltransferase PRMT9 attenuates MAVS activation through arginine methylation. Nat Commun.

[17] Kozawa K, Sekai M, Ohba K, Ito S, Sako H, Maruyama T (2021). The CD44/COL17A1 pathway promotes the formation of multilayered, transformed epithelia. Curr Biol.

[18] Chen H, Ren L, Ma W (2023). Mechanism of SOX10 in ferroptosis of hippocampal neurons after intracerebral hemorrhage via the miR-29a-3p/ACSL4 axis. J Neurophysiol.

[19] Shao L, Zhang X, Yao Q (2020). The F-box protein FBXO11 restrains hepatocellular carcinoma stemness via promotion of ubiquitin-mediated degradation of Snail. FEBS Open Bio.

[20] Ma Y, Deng F, Li P, Chen G, Tao Y, Wang H (2018). The tumor suppressive miR-26a regulation of FBXO11 inhibits proliferation, migration and invasion of hepatocellular carcinoma cells. Biomed Pharmacother.

[21] Stricher F, Macri C, Ruff M, Muller S (2013). HSPA8/HSC70 chaperone protein: structure, function, and chemical targeting. Autophagy.

[22] Bonam SR, Ruff M, Muller S. HSPA8/HSC70 in immune disorders: a molecular rheostat that adjusts chaperone-mediated autophagy substrates. Cells. 2019;8(8). 10.3390/cells8080849 PMC6721745 31394830

[23] Zhang Y, Qiao X, Liu L, Han W, Liu Q, Wang Y (2022). Long noncoding RNA MAGI2-AS3 regulates the H(2)O(2) level and cell senescence via HSPA8. Redox Biol.

[24] Kang R, Tang D, Tang D (2019). Heat shock proteins: endogenous modulators of ferroptosis. Ferroptosis in health and disease.

[25] Wang Y, Zhao M, Zhao L, Geng Y, Li G, Chen L (2023). HBx-induced HSPA8 stimulates HBV replication and suppresses ferroptosis to support liver cancer progression. Cancer Res.

[26] Wu Z, Geng Y, Lu X, Shi Y, Wu G, Zhang M (2019). Chaperone-mediated autophagy is involved in the execution of ferroptosis. Proc Natl Acad Sci USA.

[27] Liu J, Kuang F, Kroemer G, Klionsky DJ, Kang R, Tang D (2020). Autophagy-dependent ferroptosis: machinery and regulation. Cell Chem Biol.

[28] Szewczyk MM, Ishikawa Y, Organ S, Sakai N, Li F, Halabelian L (2020). Pharmacological inhibition of PRMT7 links arginine monomethylation to the cellular stress response. Nat Commun.

[29] Hassn Mesrati M, Syafruddin SE, Mohtar MA, Syahir A. CD44: a multifunctional mediator of cancer progression. Biomolecules. 2021;11(12). 10.3390/biom11121850 PMC8699317 34944493

[30] Naor D, Nedvetzki S, Golan I, Melnik L, Faitelson Y (2002). CD44 in cancer. Crit Rev Clin Lab Sci.

[31] Jang JH, Kim DH, Lim JM, Lee JW, Jeong SJ, Kim KP (2020). Breast cancer cell-derived soluble CD44 promotes tumor progression by triggering macrophage IL1beta production. Cancer Res.

[32] Muller S, Sindikubwabo F, Caneque T, Lafon A, Versini A, Lombard B (2020). CD44 regulates epigenetic plasticity by mediating iron endocytosis. Nat Chem.

[33] Qi A, Wang C, Ni S, Meng Y, Wang T, Yue Z, et al. Intravesical mucoadhesive hydrogel induces chemoresistant bladder cancer ferroptosis through delivering iron oxide nanoparticles in a three-tier strategy. ACS Appl Mater Interfaces. 2021. 10.1021/acsami.1c14944 34714617

[34] Liu T, Jiang L, Tavana O, Gu W (2019). The deubiquitylase OTUB1 mediates ferroptosis via stabilization of SLC7A11. Cancer Res.

