# Supplementary figures and images for: Arginine methylation of HSPA8 by PRMT9 inhibits ferroptosis to accelerate hepatitis B virus-associated hepatocellular carcinoma progression
Source: J Transl Med. 2023 Sep 15;21:625. doi: 10.1186/s12967-023-04408-9 (PMC10503172; doi:10.1186/s12967-023-04408-9)

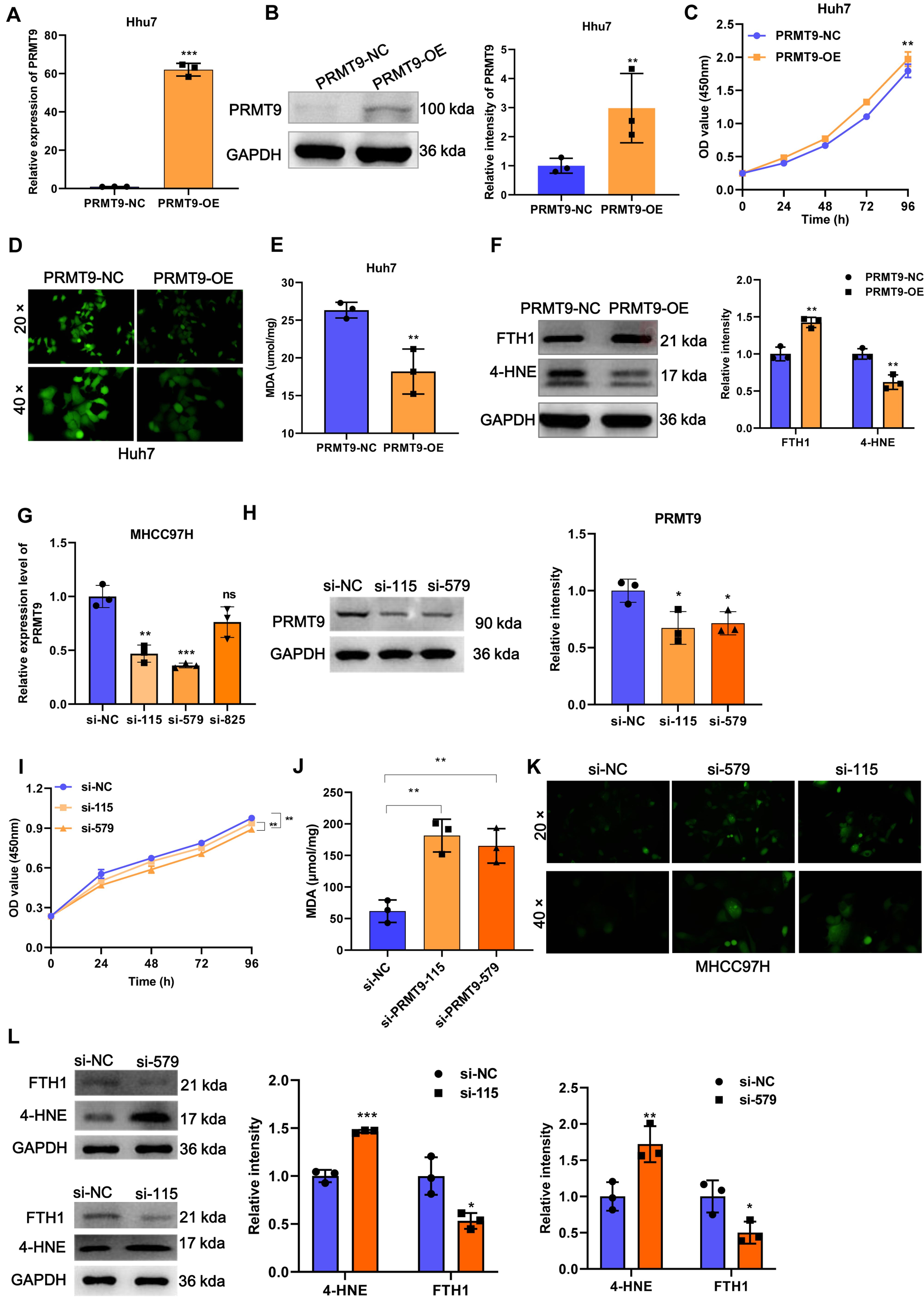

Supplement: Supplementary file 1 — Additional file 1: Figure S1. PRMT9 regulates ferroptosis in HCC cells. The overexpression efficiency of PRMT9 at mRNA (A) and protein level (B) in Hhu7 cells was detected by RT-qPCR and WB, respectively. The proliferation (C), ROS accumulation (D), MDA content (E), and ferroptosis markers expression (4-HNE and FTH1) (F) of Hhu7 cells after overexpression of PRMT9 was detected by CCK8, DCFH-DA kit, MDA detection kit, and WB, respectively. The knockdown efficiency of PRMT9 at mRNA (G) and protein level (H) in MHCC97H cells was detected by RT-qPCR and WB, respectively. The proliferation (I), MDA content (J), ROS accumulation (K), and ferroptosis markers expression (4-HNE and FTH1) (L) of MHCC97H cells after knockdown of PRMT9 was detected by CCK8, MDA detection kit, DCFH-DA kit, and WB, respectively. CCK-8 assays were performed in sextuplicate, and the remaining assays were triplicate, t test for two groups, ANOVA following Tukey’s test for three and four groups, ns p > 0.05,* p < 0.05, ** p < 0.01, *** p < 0.01 [file 12967_2023_4408_MOESM1_ESM.tif]

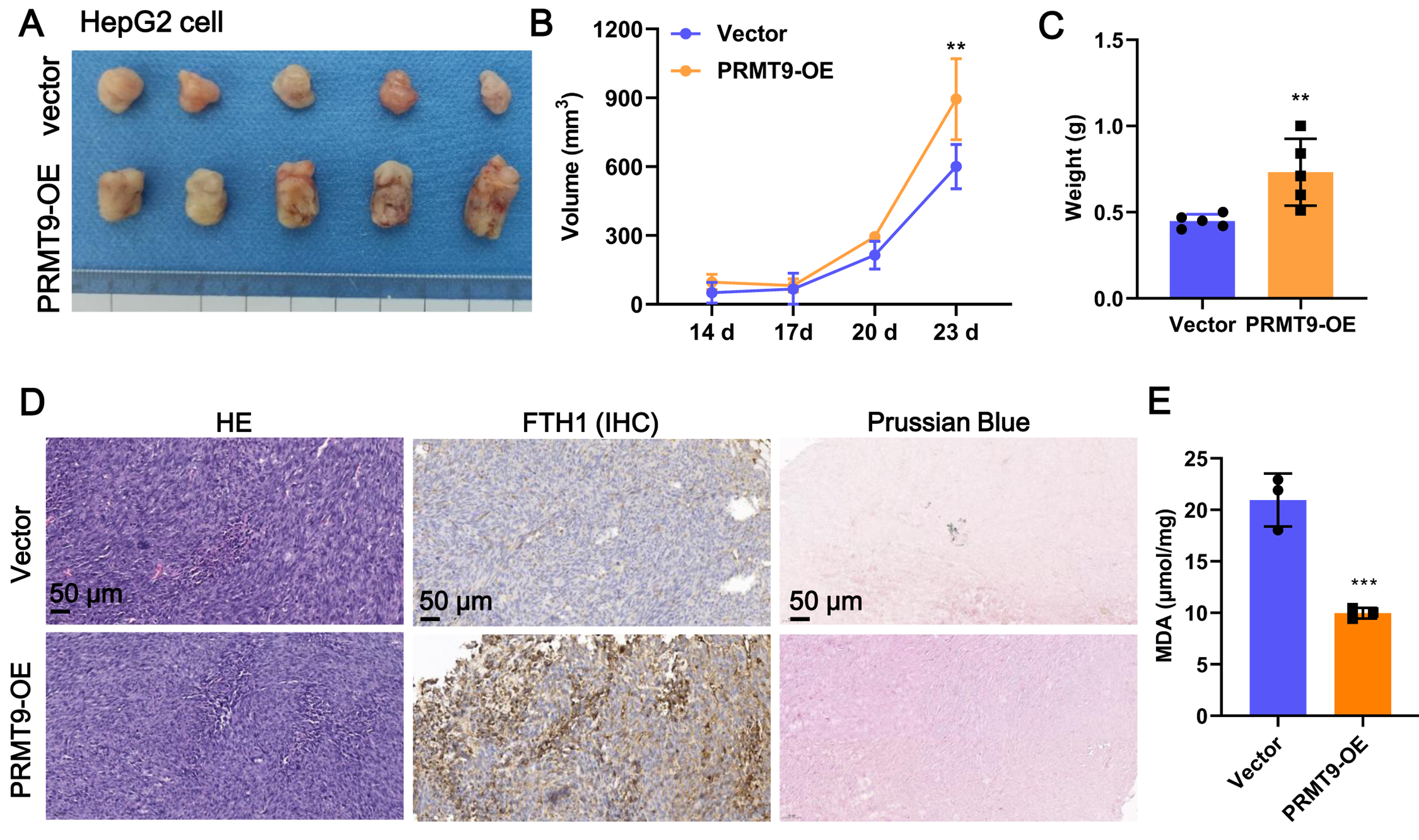

Supplement: Supplementary file 2 — Additional file 2: Figure S2. Overexpression of PRMT9 promotes HCC in mice. (A) Representative ex vivo images of tumor tissues. n = 5. (B) Tumor volume growth curves. n = 5. (C) Dissected tumor weights. n = 5. (D) Representative images of HE staining, IHC staining of FTH1 antibody, Prussian blue staining. n = 3. (E) MDA content in tumor tissues. n = 3. ** p < 0.01, *** p < 0.001 [file 12967_2023_4408_MOESM2_ESM.tif]

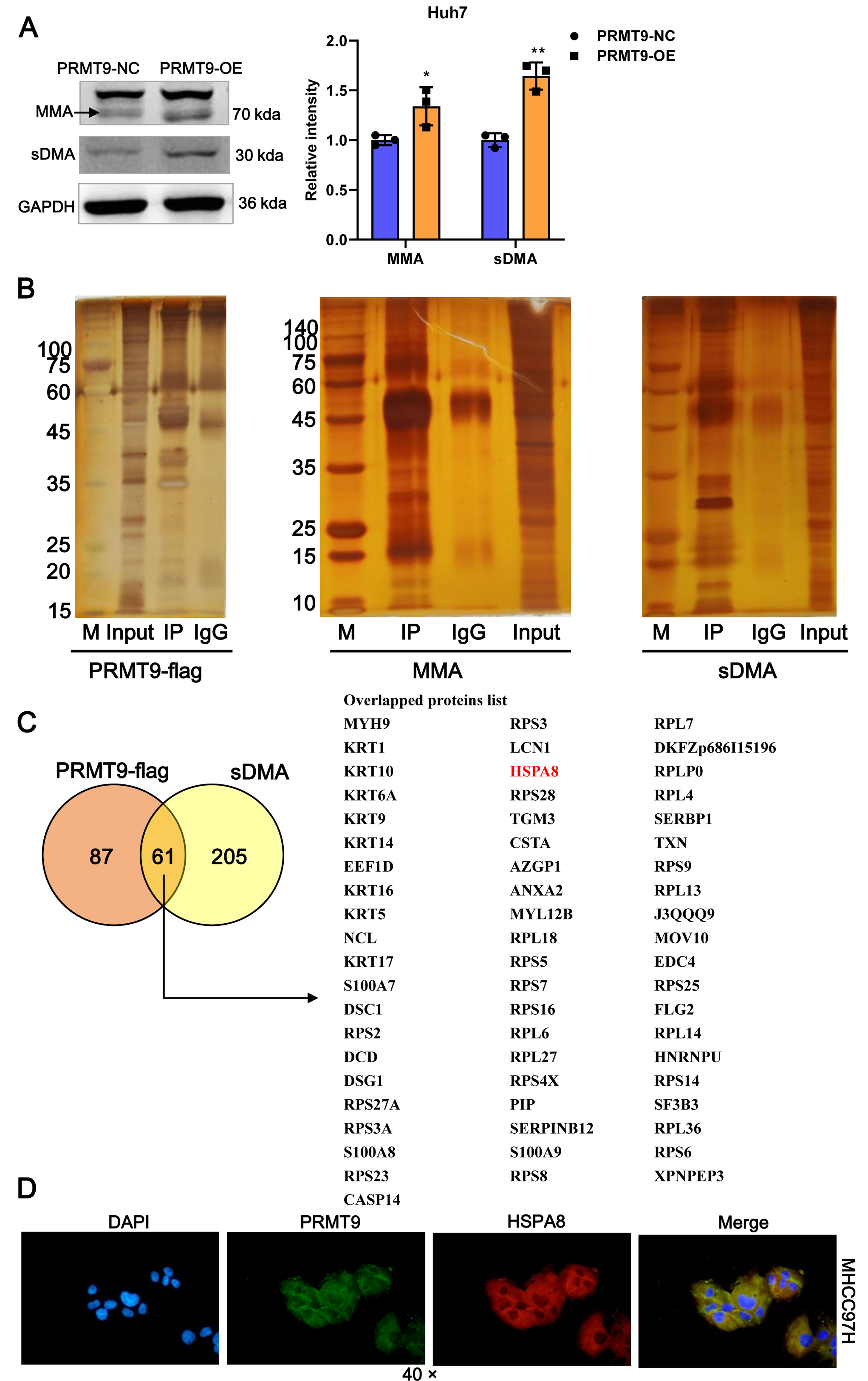

Supplement: Supplementary file 3 — Additional file 3: Figure S3. PRMT9 targets HSPA8 in HCC cells. (A) The protein expression of MMA and sDMA in Hhu7 cells after overexpression of PRMT9 was detected by WB. n = 3, t test, * p < 0.05, ** p < 0.01. (B) Flag-tag purification of the PRMT9 protein complex from Hhu7 cells, MMA co-immunoprecipitated protein complex, and sDMA co-immunoprecipitated protein complex, these eluted protein complex was separated by SDS–PAGE and silver-stained, and then identified by mass spectrometry. (C) A total of 61 proteins was overlapped by immunoprecipitation of PRMT9-flag and sDMA. (D) The colocalization between PRMT9 and HSPA8 was assessed by IF assay in MHCC97H cells. [file 12967_2023_4408_MOESM3_ESM.tif]

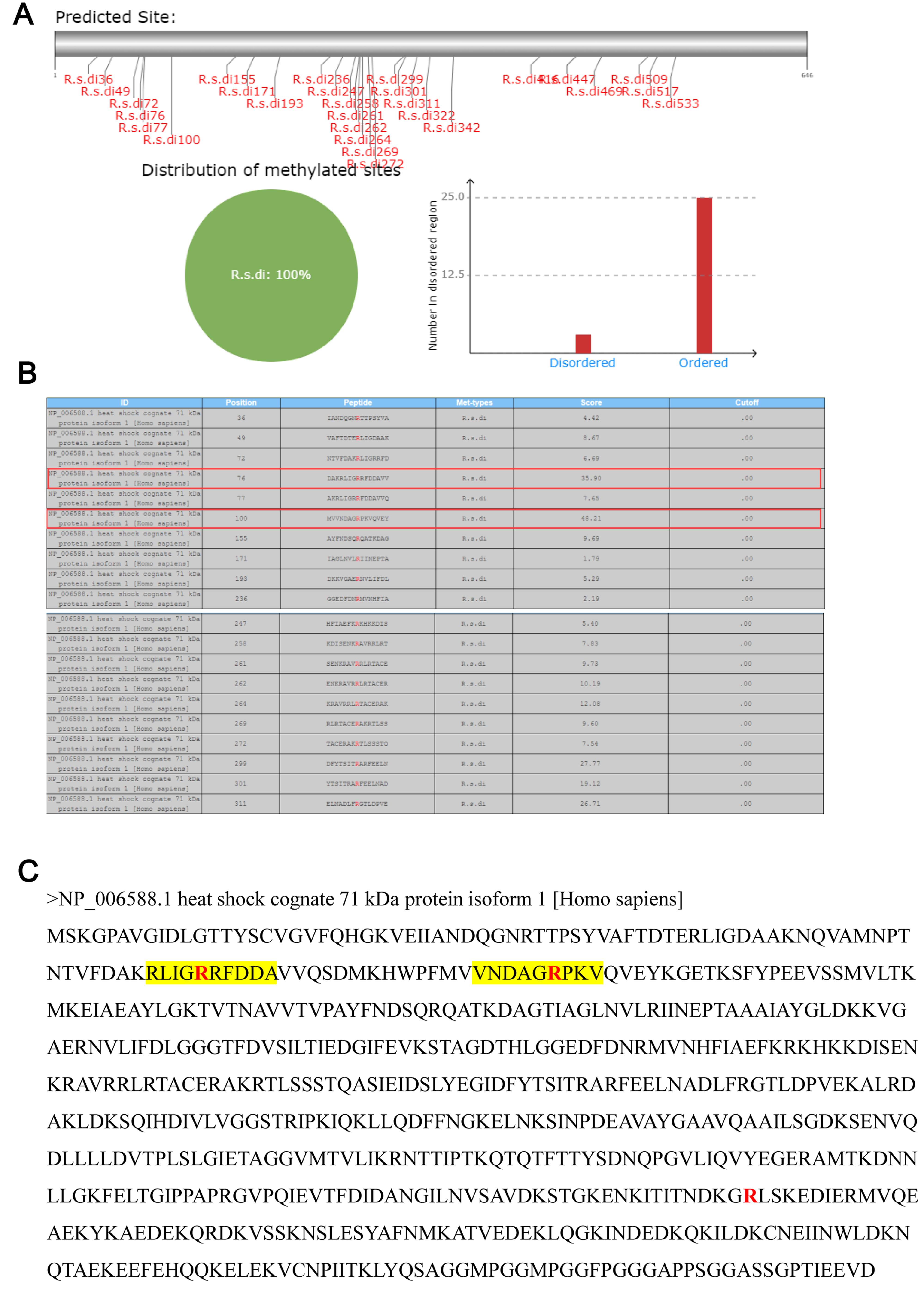

Supplement: Supplementary file 4 — Additional file 4: Figure S4. The potential arginine residues on HSPA8 that are modified by PRMT9. The predicted sDMA modification site (A) and predicted score (B) of HSPA8 using the Protein Methylation Prediction Database. (C) Amino acid sequence of HSPA8 protein. The yellow background marks the sequence for the validation site, mutating R76 and R100 to R76K and R100K, respectively. [file 12967_2023_4408_MOESM4_ESM.tif]
