# Supplementary material for: Arginine methylation of HSPA8 by PRMT9 inhibits ferroptosis to accelerate hepatitis B virus-associated hepatocellular carcinoma progression
Source: J Transl Med. 2023 Sep 15;21:625. doi: 10.1186/s12967-023-04408-9 (PMC10503172; doi:10.1186/s12967-023-04408-9)
Supplement: Supplementary file 5 — Additional file 5: Table S1. The primers used in this study. [file 12967_2023_4408_MOESM5_ESM.docx]

Table S1. The primers used in this study.

| Gene | Primers (5’ - 3’) |
| --- | --- |
| GAPDH-F | AGAAGGCTGGGGCTCATT |
| GAPDH-R | AGAAGGCTGGGGCTCATT |
| HSPA8-F | TGCTGTGGACAAGAGTACGG |
| HSPA8-R | AGCTTCCTGGACCATACGTT |
| CD44-F | CTACAGCATCTCTCGGACGG |
| CD44-R | ACTGCAGGTCTCAAATCCGA |

| PRMT9-F | TGGAGAAGGAATTGTGGAGAGT |
| --- | --- |
| PRMT9-R | CACATTCTACTGCCATCCCAAA |
| HBX-F | GCCCACCAAATATTGCCCAA |
| HBX-R | CCCAACTCCTCCCAGTCTTT |
| shRNA-PRMT9-1 | GCACACTTGGGCAGGTTAAAC |
| shRNA-PRMT9-2 | CCATATCATGAAGGCTTTAAA |
| shRNA-PRMT9-3 | GAACGTACTCTTGGATTAAAT |
| shRNA-PRMT9-NC | CCTAAGGTTAAGTCGCCCTCG |

| si-NC | UUCUCCGAACGUGUCACGUTT  ACGUGACACGUUCGGAGAATT | |
| --- | --- | --- |
| si-579 | GUGCUAAUUGUGAAAAGUATT  UACUUUUCACAAUUAGCACTT | |
| si-115 | GCACUUUAUCAUGCUUAAUTT  AUUAAGCAUGAUAAAGUGCTT | |
| si-825 | | CUAAAAAGCCUGAUAAGAUTT  AUCUUAUCAGGCUUUUUAGTT |

Note: F means forward primers, R means reverse primers, RT means reverse transcription
